# Variations in the Metabolome of Unaged and Aged Beef from Black-and-White Cows and Heifers by ^1^H NMR Spectroscopy

**DOI:** 10.3390/foods12040785

**Published:** 2023-02-13

**Authors:** Greta Bischof, Edwin Januschewski, Franziska Witte, Nino Terjung, Volker Heinz, Andreas Juadjur, Monika Gibis

**Affiliations:** 1 German Institute of Food Technologies (DIL e.V.), Prof.-v.-Klitzing-Str. 7, 49610 Quakenbrück, Germany; 2 Department of Food Material Science, Institute of Food Science and Biotechnology, University of Hohenheim, Garbenstr. 25, 70599 Stuttgart, Germany

**Keywords:** dry-aged, wet-aged, bovine, maturation, ripening, aging time, aging type, meat

## Abstract

(1) Background: The selection of raw material and the postmortem processing of beef influence its quality, such as taste. In this study, the metabolome of beef from cows and heifers is examined for differences during aging. (2) Methods: Thirty strip loins from eight heifers and seven cows (breed code: 01–SBT) were cut into ten pieces and aged for 0, 7, 14, 21 and 28 days. Samples from the left strip loins were wet-aged in vacuum, while samples from right strip loins were dry-aged at 2 °C and 75% relative humidity. The beef samples were extracted with methanol–chloroform–water, and the polar fraction was used for ^1^H NMR analysis. (3) Results: The PCA and OPLS-DA showed that the metabolome of cows and heifers varied. Eight metabolites revealed significant differences (*p* < 0.05) in the samples from cows and heifers. The aging time and aging type of beef also affected the metabolome. Twenty-eight and 12 metabolites differed significantly (*p* < 0.05) with aging time and aging type, respectively. (4) Conclusions: The variations between cows and heifers and aging time affect the metabolome of beef. By comparison, the influence of aging type is present but less pronounced.

## 1. Introduction

The selection of raw material has an important influence on beef quality parameters, such as taste. In addition to the specification of the raw material, such as breed, age, or sex, extrinsic factors, such as the aging method, also affect the quality of beef [1]. Traditional dry-aging without any packaging and wet-aging in vacuum are the two conventional aging methods [2].

The aging method (aging type and aging time) affects the metabolome of beef [3], which contains flavor precursors and components. Amino acids, such as leucine or isoleucine, were reported to correlate with the aging time [4]. The aging type also influenced metabolites in beef, such as tryptophan, glutamate [5], lactic acid, and anserine [6]. In addition to the aging method, the metabolome of beef is also influenced by intrinsic factors, such as breed or sex [7,8].

According to the definition of the European classification system [9], heifers and cows are both adult females, whereby the cow, in contrast to the heifer, has already calved at least once. Heifers are inseminated as soon as they have reached 2/3 of their final weight. Depending on the breed, this is the case after 15–25 months. One third of all heifers are older than 30 months at first calving [10]. If heifers are not used for milk production, they are fattened until the desired weight class and fatness is reached, and then slaughtered [11]. Watanabe, et al. (2004) [12] reported that steers’ age at slaughter (15, 25, and 35 months old) affects the concentration of amino acids, such as aspartic acid, glutamine, glycine, alanine, valine, and leucine, as well as the concentration of dipeptides carnosine and anserine in beef. Furthermore, Cho, et al. (2013) [13] analyzed Hanwoo cows in three different age groups (<60; 72–96 and >108 months) and found significantly (*p* < 0.05) higher contents of alanine (15.89 ± 0.37 to 13.68 ± 0.28 and 13.76 ± 0.33 µmol/g), leucine (0.66 ± 0.02 to 0.54 ± 0.01 and 0.55 ± 0.01 µmol/g), methionine (0.70 ± 0.03 to 0.63 ± 0.03 and 0.63 ± 0.02 µmol/g), and phenylalanine (0.87 ± 0.02 to 0.74 ± 0.03 and 0.69 ± 0.01 µmol/g) in beef from cows <60 months old than in beef from older cows. The analysis of wet-aged beef from heifers (27–40 months) and cows (103–150 months) over the aging time showed a trend in samples due to the aging time along the PC 1 axis, and due to heifers or cows along the PC 2 axis in principal component analysis (PCA) [4]. These results suggest an influence of the aging time and cattle type (cow or heifer) on the metabolome, with the limitation that the authors did not analyze any unaged samples from cows and heifers. The authors reported higher amounts of glutamine, fumaric acid, betaine, anserine, and alanine in beef from heifers than in beef from cows based on the PCA results.

The objective of this study is to analyze the effects of differences between cows and heifers and the effects of the aging method on the metabolome of fresh, dry-aged, and wet-aged beef. It is intended to determine which influence on the metabolome of beef is more pronounced and statistically separable. We hypothesize that the metabolic differences between cows and heifers are more pronounced than the effect of the aging type.

## 2. Materials and Methods

### 2.1. Raw Material

Eight heifers and seven cows of the ‘Black-and-White’ breed (‘*Holstein-Schwarzbunte*’ in German; code: 01-SBT) from German high-input production systems were used. The heifers were between 18 and 23 months old, while the cows ranged from 44 to 85 months old. All heifers and cows were graded from 2 to 3 (low to medium fat cover) and O (average muscle fullness) according to the European Union classification system EUROP [9]. The right and left bone-in strip loin (*M. longissimus thoracis et lumborum*) were taken from the 5th rib to 6th lumbar vertebra five days postmortem.

### 2.2. Beef Aging

Ten pieces, each with 8 cm thickness, were cut from each strip loin cranial to caudal, two pieces per time point (0, 7, 14, 21, 28 days). Samples were aged in four aging runs (R1 to R4) with pieces from three or four animals per run. Aging was performed in an aging chamber (AT 1-6/E, Autotherm Ludwig Brümmendorf GmbH & Co. KG, Waxweiler, Germany) with R1, 2.0 ± 1.2 °C and 77.0 ± 7.3% relative humidity; R2, 2.0 ± 1.2 °C and 77.3 ± 7.4% relative humidity; R3, 2.1 ± 1.3 °C and 78.3 ± 5.2% relative humidity; and R4, 1.7 ± 1.3 °C and 78.0 ± 8.1% relative humidity. The right bone-in strip loins were used for dry-aging without any packaging, while the left deboned strip loins were wet-aged under vacuum (<15 mbar) in polyamide/polyethylene bags (BST-090; Rolf Bayer Vacuumverpackung GmbH, Veitsbronn, Germany; oxygen permeability < 60 mL/m^2^ per 24 h per bar at 0% relative humidity and 23 °C and water permeability < 4 g/m^2^ per 24 h at 85% relative humidity and 23 °C). The surface of the dry-aged beef was trimmed approx. 1 cm on each side after aging.

### 2.3. Sample Preparation

Samples were taken from the inside of dry-aged and wet-aged beef for NMR analysis. According to the method of Bischof, et al. (2020) [14], 200 mg of samples (raw meat, wet mass) were mixed with 0.4 mL methanol (99.9%; Lab-Scan, Bangkok, Thailand) and 0.0875 mL ultrapure water (Milli-Q Organex-Q System, Merck, Millipore, Darmstadt, Germany). After homogenization (30 s at 6 m/s; two stainless steel beads with 3.2 mm diameter; Bead Ruptor Elite Bead Mill Homogenisator; OMNI International, Kennesaw, GA, USA), 0.4 mL chloroform (99.5%; Lab-Scan, Bangkok, Thailand) and 0.2 mL ultrapure water were added, followed by a second homogenization step under the same conditions. Samples were cooled on ice for 10 min and then centrifuged (6900× *g*, 20 min, 4 °C; Hettich Universal 320 R, Hettich GmbH & Co. KG, Tuttlingen, Germany). The upper phase (600 µL) was dried under vacuum (Eppendorf Concentrator plus, Eppendorf AG, Hamburg, Germany). The pellets were then resuspended in 800 µL of 100 mM sodium phosphate buffer containing NaH_2_PO_4_ (sodium dihydrogen phosphate monohydrate, 99.5%; AppliChem, Darmstadt, Germany), Na_2_HPO_4_ (disodium hydrogen phosphate, 99.3%; AppliChem, Darmstadt, Germany), 0.01% NaN_3_ (≥99%; AppliChem, Darmstadt, Germany), 10% D_2_O (99.9%D; Sigma Aldrich, St. Louis, MO, USA), and 80 μL internal standard (0.33% 3-(trimethylsilyl)propionic acid-2,2,3,3-d4 (99%; abcr GmbH, Karlsruhe, Germany), 100 mM imidazole (≥99%; Fluka Analytical, Munich, Germany), 11 mM maleic acid (≥99%; AppliChem, Darmstadt, Germany). The pH was adjusted to 7 (BTpH, Bruker Biospin GmbH, Rheinstetten, Germany), and 600 µL of the samples were transferred to an NMR tube (5 mm; Deutero GmbH, Kastellaun, Germany). The lower phase was discarded because preliminary experiments and further literature [4] had shown that the analysis of the lower phase did not reveal detectable changes by ^1^H NMR spectroscopy, possibly due to the overlay of signals in spectra or to the different marbling of the samples.

### 2.4. NMR Spectroscopy

The ^1^H NMR measurement was performed using a Bruker 400 MHz Ascend NMR spectrometer (Bruker Biospin GmbH, Rheinstetten, Germany) and Topspin 3.6 software (Bruker Biospin GmbH). The measurement parameters were selected as follows: pulse program, noesygppr1d; temperature, 300 K; number of points, 65 k; number of scans, 128; spectral width, 8.403 kHz; acquisition time, 3.9 s; and presaturation field strength for water suppression, 25 Hz. Metabolite identification was performed using the NMR database (Bruker Biospin GmbH), according to references [4,5,14,15] and self-measured standard components. All NMR spectra were normalized and scaled to the signal of maleic acid as internal standard compound.

### 2.5. Data Analysis

#### 2.5.1. Non-Targeted Analysis

Matlab R2018a software (The Mathworks, Natick, MA, USA) was used for data analysis. The number of buckets and the spectral ranges were varied for optimization, and the bucket number and spectral ranges with the best results in terms of separation of cattle type, aging time, and aging type in the PCA were used for further analysis. In the range from 0.50 to 4.67 ppm, most of the signals were in the ^1^H NMR spectra of the beef extract. Regarding the aging time and cattle type, 250 buckets of the same size were fixed from 0.50 to 4.67 ppm in the ^1^H NMR spectra. Univariate scaling was performed with all buckets forming the basis for the statistical analysis. A PCA was used to analyze the NMR spectra. An orthogonal projection to latent structures discriminant analysis (OPLS-DA; 25-fold cross-validation) was utilized to analyze the NMR spectra for the cattle type (cow or heifer), and partial least squares regression (PLS-R; 10-fold cross-validation) was used to analyze the aging time. Regarding the aging type, the ^1^H NMR spectra were divided into 200 equally sized buckets ranging from 1.1 to 9.0 ppm. The solvent signal (4.7 to 5.2 ppm) and the region (6.8 to 8.0 ppm) were excluded. After univariate scaling and PCA, a linear discriminant analysis (LDA; 10-fold cross-validation) was performed.

#### 2.5.2. Targeted Analysis

**Figure 1:**
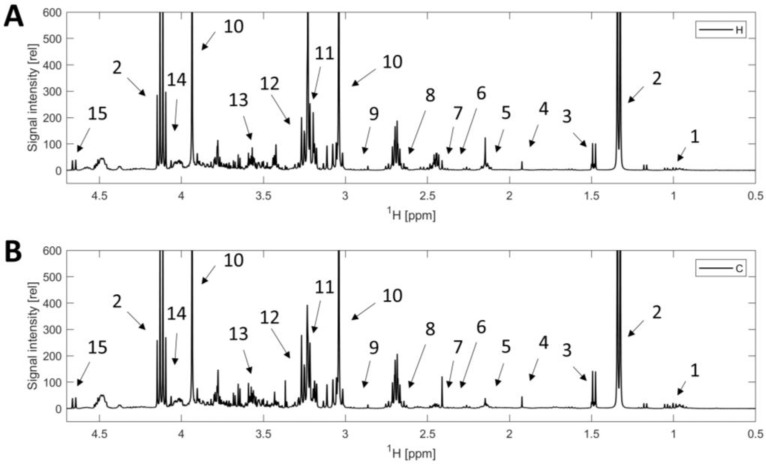
^1^H NMR spectra of beef extract in 90% H_2_O + 10% D_2_O. (**A**): beef sample from heifer. (**B**): beef sample from cow. 1. Isoleucine, leucine, valine; 2. Lactic acid; 3. Alanine; 4. Acetic acid; 5. Glutamine; 6. Glutamate; 7. Succinic acid; 8. Methionine; 9. Aspartate; 10. Creatine; 11. Carnitine; 12. Betaine; 13. Glycine; 14. Creatinine; 15. β glucose.

The metabolite qualification and quantification were performed as described in our previous studies [6,14]. The calculation of metabolite concentrations was based on manually set integrals of the signals shown in Figure 1 and Table A1. The equation is

(1) $$P_{analyte}=\frac{I_{analyte}}{I_{standard}}\cdot \frac{N_{standard}}{N_{analyte}}\cdot \frac{M_{analyte}}{M_{standard}}\cdot \frac{m_{standard}}{m_{analyte}}\cdot P_{standard}$$

with *P* (purity), *I* (integral of signal), *N* (number of protons in signal), *M* (molecular mass), and *m* (mass).

Concentrations of metabolites were statistically analyzed using two generalized linear mixed effect models to identify the influence of fixed and random effects.

Model 1. Concentration of metabolite~1 + aging time ∗ (cattle type + aging type) + (1|Animal) + (1|aging run).

Model 2. Concentration of metabolite~1 + cattle type + (1|animal).

The models were simplified by backward selection, removing fixed effects that were not significant (*p* > 0.05). The calculated metabolite concentrations were not normally distributed (proven with Shapiro–Wilk test); therefore, nonparametric tests were used for statistical analysis. Spearman’s rank–order correlation coefficient r_SP_ (α = 0.05), the Friedman’s test, and Kruskal–Wallis test with post-hoc Bonferroni test (α = 0.05) were calculated. The Spearman’s rank–order correlation coefficient ranges from −1 to 1, indicating highly negative (r_SP_ = −1), highly positive (r_SP_ = +1), or no correlation (r_SP_ = 0). A pathway analysis as over-representation analysis was performed using MetaboAnalyst 5.0 (https://www.metaboanalyst.ca; accessed on 14 December 2022) with the *Bos Taurus* library.

## 3. Results and Discussion

### 3.1. Influence of Aging Time on Beef Metabolome

Strip loins from 15 female cattle (eight heifers and seven cows) were wet- and dry-aged up to 28 days, extracted, and analyzed by ^1^H NMR spectroscopy to obtain information on the metabolome and metabolites that differed according to the aging time and aging type.

**Figure 2:**
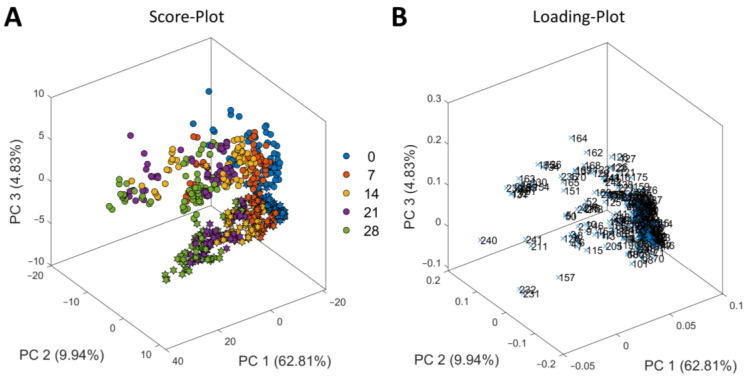
Principal component analysis (PCA) based on the ^1^H NMR spectra of unaged and aged beef samples. (**A**) Score plot. The samples from heifers are marked with circles and from cows with hexagrams. The aging time of samples was 0 (blue), 7 (red), 14 (yellow), 21 (purple), and 28 (green) days. (**B**) Loading plot. The index numbers indicate the number of buckets (Table S1).

First, PCA for exploratory data analysis was performed on the ^1^H NMR spectra (Figure 2 and Figure S1). The score plot (Figure 2A) showed that the samples shift along PC 1 due to aging time. The additional loading plot (Figure 2B) demonstrated that the buckets in the range of 0.75 to 2.35 ppm, 2.80 to 2.90 ppm, 3.60 to 4.00 ppm, and 4.22 to 4.36 ppm were crucial for this trend (Table S1). Signals from amino acids such as valine, leucine, and isoleucine, and signals from acetic acid and lactic acid were present in these ranges. The shift along PC 2 is due to the animal’s individual differences in the ^1^H NMR spectra. The samples with very low PC 2 values were from two different heifers that show characteristic differences in all their ^1^H NMR spectra (0 to 28 days, dry-aged and wet-aged) compared to the other spectra of the heifer samples (Figure S2). The loadings for PC 2 (Table S1) showed that the variations in the ^1^H NMR spectra ranged from 1.30 to 1.35 ppm, 2.55 to 2.80 ppm, 4.10 to 4.15 ppm, and 4.45 to 4.52 ppm. Signals from sugars, such as glucose, and dipeptides, such as carnosine and anserine, are present in these ranges. The information and specifications from the German slaughterhouse did not explain these outliers, therefore, the reason for these variations between the different heifers is still unknown.

**Figure 3:**
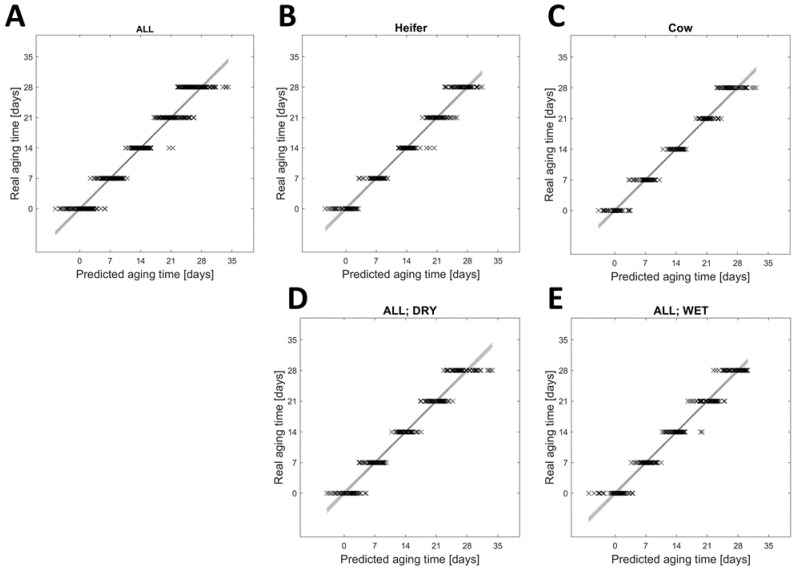
Partial least squares regression (PLS-R). The PLS-R is based on samples in various groups: all (**A**), only those from heifers (**B**), only those from cows (**C**), only unaged and dry-aged samples (**D**), and only unaged and wet-aged samples (**E**). The PLS-Rs were performed with 10-fold cross-validation. The dotted lines represent the 95% confidence interval. Parameters for the different models: (**A**) R^2^ = 0.956, RMSEC = 2.088, Q^2^ = 0.940, RMSECV = 2.420. (**B**) R^2^ = 0.966, RMSEC = 1.816, Q^2^ = 0.949, RMSECV = 2.236. (**C**) R^2^ = 0.973, RMSEC = 1.630, Q^2^ = 0.952, RMSECV = 2.161. (**D**) R^2^ = 0.961, RMSEC = 1.948, Q^2^ = 0.942, RMSECV = 2.383. (**E**) R^2^ = 0.966, RMSEC = 1.839, Q^2^ = 0.947, RMSECV = 2.282.

A statistical analysis of the ^1^H NMR spectra using PLS-R (Figure 3) supported the PCA results that the aging time of beef strongly influenced the metabolome. The PLS-R was performed on all (A), heifer only (B), cow only (C), dry-aged only (D), and wet-aged samples only (E). The R^2^ of regression was >0.95 and the cross-validated R^2^ (Q^2^) was >0.94 in all these five groups, implying that the effect of aging time on the metabolome is present in all samples and independent of the cattle type (cow or heifer) or the aging type. The RMSE of calibration ranged from 1.630 to 2.088 and the RMSE of cross-validation ranged from 2.161 to 2.420. In this case, the RSME can be related to the aging days and showed that the predicted aging day can differ by ± 2.4 days. The prediction was slightly better when the PLS-R was separated for cow (RSMECV = 2.161) and heifer (RSMECV = 2.236). The VIP scores (Table S2 and Figure S4) of all five PLS-Rs demonstrated that the buckets 28 to 31, 163 to 166, and 183 to 217 played an important role for this analysis (VIP score > 1). The highest VIP scores (2.5 to 6.9) were observed in the buckets associated with signals from lactic acid, isoleucine, leucine, and valine. The ppm area of these buckets is consistent with the ppm area determined by the PCA loadings.

Further, these results are also reflected in the statistical analysis of the metabolites. The generalized linear mixed effect model 1 demonstrated that the concentrations of 28 metabolites were significantly affected (*p* < 0.05) by the aging time (Table S3). The aging run (R1 to 4) as a random effect showed no influence on the metabolites. Furthermore, the concentration of the seven metabolites isoleucine (0.21 ± 0.05 to 1.09 ± 0.26 µmol/g), leucine (0.32 ± 0.09 to 1.83 ± 0.42 µmol/g), phenylalanine (0.53 ± 0.04 to 1.26 ± 0.19 µmol/g), tryptophan (0.13 ± 0.01 to 0.27 ± 0.04 µmol/g), tyrosine (0.30 ± 0.06 to 1.11 ± 0.22 µmol/g), valine (0.36 ± 0.08 to 1.82 ± 0.42 µmol/g), and hypoxanthine (1.20 ± 0.30 to 3.97 ± 0.76 µmol/g) increased and correlated positively (r_SP_ ≥ 0.88), while the concentration of the metabolite IMP (3.43 ± 0.33 to 1.25 ± 0.38 µmol/g) decreased and correlated negatively (r_SP_ = −0.92) with aging time (Table A1). The results of PCA, PLS-R, and the generalized linear mixed effect model showed three common metabolites (isoleucine, leucine, and valine) that played an important role in beef during aging. In our previous study, we found the same metabolites as marker metabolites of aging time in beef from Simmental young bulls [6]. Furthermore, these results are in agreement with the literature [7,16,17,18]. The increase of amino acids during aging of beef may be related to the protein degradation postmortem and during aging [19], and therefore may be an indicator of tenderness of beef. In addition, amino acids may influence the taste of beef [20].

### 3.2. Differences in the Metabolome of Beef between Cows and Heifers

Analysis of differences in the beef metabolome between cows and heifers is important to separate differences that depend on cattle type from changes related to the aging method. When looking for biomarkers related to the aging method, the potential biomarkers may be present in heifers but not in cows, or vice versa. Capturing these factors could later open up the possibility of verifying the authenticity of an expensive dry-aged product in particular. However, this will require further investigations in the future.

The PCA in Figure 2 also demonstrated the grouping of heifer and cow samples along the PC 3, indicating differences in the ^1^H NMR spectra (Figure 1) and thus in the metabolome of beef samples due to the cattle type (heifer vs. cow). This trend in PCA is consistent with the findings of Kodani, et al. (2017) [4] who have already shown the clustering of the ^1^H NMR spectra of cow and heifer samples in PCA.

**Figure 4:**
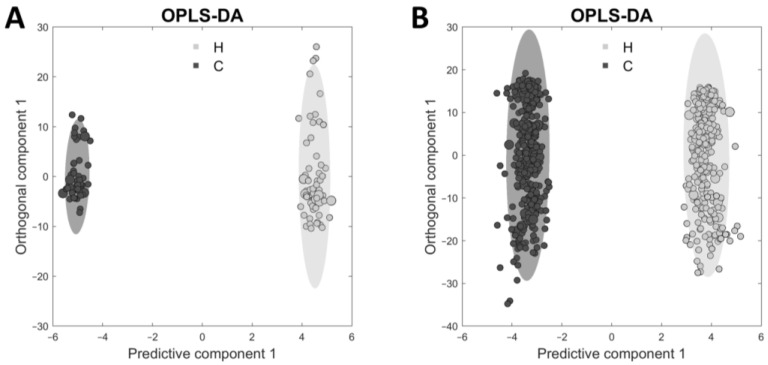
Orthogonal projections to latent structures discriminant analysis (OPLS-DA) of ^1^H NMR spectra of beef samples from cows and heifers. (**A**): OPLS-DA with 10-fold cross-validation and an accuracy of 99.1% from 0-days beef samples from cows (dark gray) and heifers (light gray). (**B**): OPLS-DA with 25-fold cross-validation and an accuracy of 99.8% from all samples (0 to 28 days; dry-aged and wet-aged) from cows (dark gray) and heifers (light gray). The ellipses demonstrate the 95% confidence interval. The coefficient of determination is R^2^ = 0.998 (**A**) and R^2^ = 0.990 (**B**). The cross-validated R^2^ is Q^2^ = 0.985 (**A**) and Q^2^ = 0.985 (**B**).

The statistical analysis of the ^1^H NMR spectra was performed using the supervised method OPLS-DA, since this supervised method includes the class memberships of samples and thus allows a better discrimination of the samples. Based on the results of PCA with a good separation of NMR spectra of cow and heifer samples, the often-mentioned problem of overfitting when using OPLS-DA can be excluded in this case. The OPLS-DA (Figure 4) revealed models between cows and heifers in both unaged samples (0 days) and all samples (0 to 28 days) with accuracies of 99.1% and 99.8%, respectively. The R^2^ and Q^2^ additionally demonstrated that over 0.99 of the variances were determined by the model, and 0.98 of the variances were confirmed by the cross-validated R^2^ (Q^2^), resulting in the validity of the OPLS-DA models. Further, the PCA and OPLS-DA showed that the differences in the metabolome between cows and heifers persisted before and during aging.

**Figure 5:**
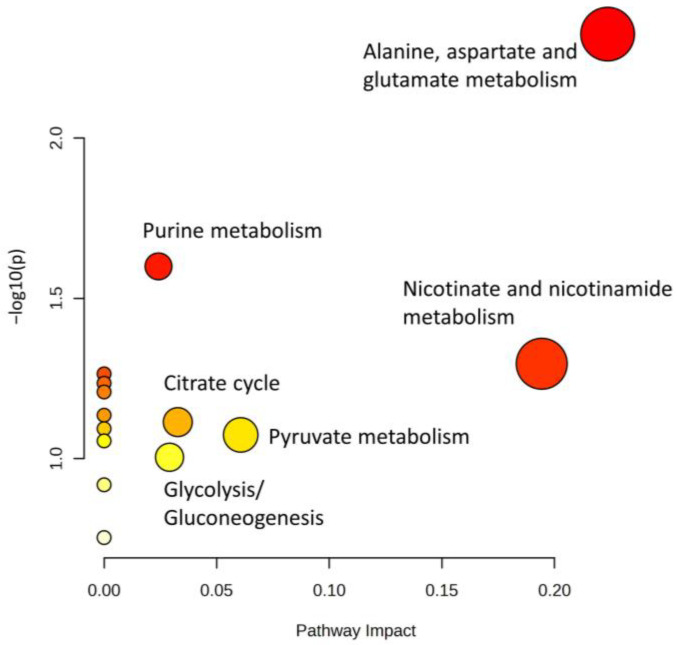
Pathway analysis by over-representation analysis. The metabolites with significant differences (*p* < 0.05, Table S3) in samples from cows and heifers were used. The radius of the circle is based on the pathway impact values, and the color of the circle corresponds to the *p*-value.

The concentrations of eight metabolites (aspartate, hypoxanthine, inosine, acetic acid, succinic acid, carnitine, niacinamide, and O-acetyl-L-carnitine) in unaged and aged beef by the cattle type (cow and heifer) were significantly (*p* < 0.05) affected. Already in unaged beef, the concentrations of the metabolites acetic acid, fumaric acid, succinic acid, carnitine, creatine, niacinamide, and O-acetyl-L-carnitine differed (*p* < 0.05) between cows and heifers (Table S3). These results indicated that the variations in the metabolome between cows and heifers were also present in unaged beef and, for some metabolites, were still present after aging up to 28 days. The generalized linear mixed effect model 1 revealed an interaction of cattle type and aging time for 17 metabolites (Table S3). The differences between cows and heifers in their metabolome may be due to the differences in slaughter age and/or changes in metabolome resulting from, or combined with, post-calving and lactation [21]. The effect of the latter on beef (cow) represents a gap in the previous literature. Watanabe, et al. (2004) [12] and Cho, et al. (2013) [13] found that the slaughter age in bulls and cows had an impact on the metabolome of beef, affecting, among other factors, the concentration of amino acids. The over-representation analysis with metabolites that were significantly different between cows and heifers of all samples (0 to 28 days, dry-aged and wet-aged) showed that the major metabolic pathways affected in beef between cows and heifers are the alanine, aspartate, and glutamate metabolism, nicotinate and nicotinamide metabolism, and purine metabolism (Figure 5).

### 3.3. Influence of Aging Type on the Metabolome

**Figure 6:**
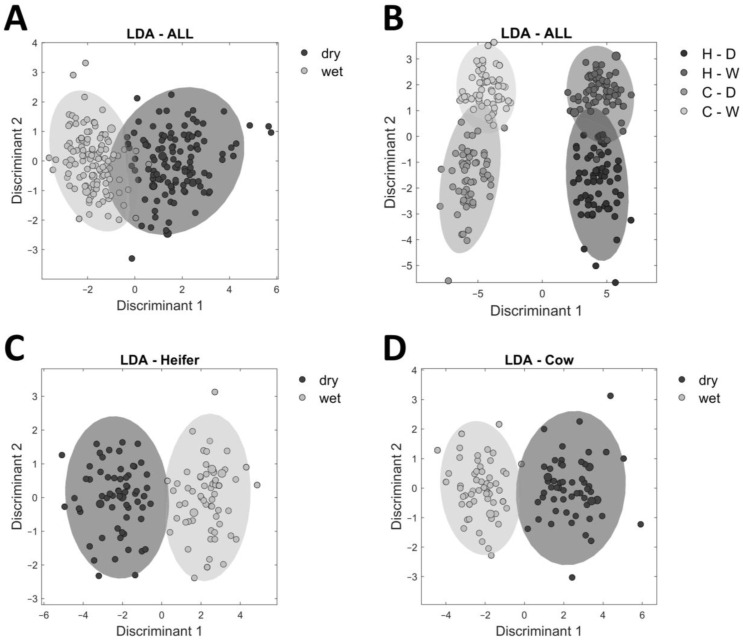
Linear discriminant analysis (LDA) of ^1^H NMR spectra of wet-aged and dry-aged samples. All ^1^H NMR spectra of samples with 21 and 28 days of aging were used. ^1^H NMR spectra of samples from cows and heifers (**A**); from cows and heifers, each grouped in dry-aged and wet-aged (**B**); from heifers (**C**); and from cows (**D**). A 10-fold cross-validation was performed. The accuracy is 93.5% (**A**), 94.9% (**B**), 98.4% (**C**), and 98.13% (**D**).

The analysis of the aging type was based on the bucketing of ^1^H NMR spectra from 1.1 to 9.0 ppm. An LDA was used as a supervised method to calculate a statistical model to distinguish the ^1^H NMR spectra of dry-aged and wet-aged samples. An LDA minimizes the within-group variances and maximizes the between-group variances to achieve better discrimination between samples [22]. The weakness of LDA is handling a large number of variables. To circumvent this weakness, a PCA (Figure S3) was performed in advance for dimensionality reduction. The model (Figure 6) included the samples of 21 and 28 days of age, since the differences between the aging types become manifest after a minimum aging time. The LDA model of all samples (21 and 28 days) in two groups (dry-aged vs. wet-aged) had an accuracy of 93.5% (A). The LDA models separated for heifer and cow samples revealed accuracies of 98.4% (C) and 98.1% (D), respectively. This observation supports the results described above that the metabolome of beef from cows and heifers is different. The LDA model in four groups demonstrated that the differences in metabolome between samples from cows and heifers were greater than the differences due to aging type, because the dry-aged and wet-aged samples overlapped in the respective cattle type (cow or heifer).

The targeted analysis of the calculated metabolites demonstrated that twelve out of 30 metabolites (glutamate, isoleucine, leucine, phenylalanine, tyrosine, tryptophan, valine, hypoxanthine, IMP, inosine, creatinine, and niacinamide) were significantly affected by the aging type (Table S3), indicating that these metabolites are potential biomarkers. An interaction between the aging time and aging type was observed for 15 metabolites (glutamate, isoleucine, leucine, phenylalanine, tyrosine, tryptophan, valine, hypoxanthine, IMP, lactic acid, betaine, carnitine, creatine, creatinine, and niacinamide) that included all twelve above-mentioned metabolites which were affected by aging type. Thus, all detected metabolites, which showed a significant effect of aging type, also were affected by aging time. This means that no potential biomarker was found that was dependent on the aging type only. Compared to our previous study using samples from Simmental young bulls, four compounds (lactic acid, anserine, fumaric acid, and O-acetyl-L-carnitine) differed significantly due to the aging type [6]. These results suggest that the breed and sex affect the metabolome and changes in metabolome during the aging of beef. Kim, et al. (2020) [23] and Setyabrata, et al. (2022) [24] analyzed 28-days dry-aged and wet-aged beef and found significant differences in the concentration of 13 and 20 amino acids, respectively. The aging type and its influence on the metabolome appear to be dependent on intrinsic factors, such as the breed, sex, and age of the cattle type. Our results indicate that the aging type of beef affects the metabolic processes in beef during aging, and thus the aging outcome of beef. Changes in metabolites such as amino acids or nucleotides can influence the flavor of beef [20].

## 4. Conclusions

The metabolome of unaged and aged beef differed in samples from cows and heifers of breed Black-and-White, and changed with aging time. It is possible to predict the aging time of dry-aged and wet-aged beef samples with a precision of ±2.4 days by PLS-R of ^1^H NMR spectra. The cattle type (cow or heifer) can be discriminated by OPLS-DA of ^1^H NMR spectra independent of aging time and aging type of beef samples. From the LDA, the influence of aging type on the metabolome of beef is less pronounced than the effect of the cattle type and aging time. The twelve observed metabolites that were affected by the aging type were also affected by the aging time. In future studies, sensory analysis will be helpful to determine whether the changes in the metabolome affect the taste and aroma of beef. Furthermore, it would also be interesting to study samples from bulls and young bulls to determine the influence of the slaughter age and sex.

## Data Availability

The datasets generated for this study are available on request to the corresponding author.

## Supplementary Materials

The following supporting information can be downloaded at: https://www.mdpi.com/article/10.3390/foods12040785/s1, Figure S1: Further display options of the calculated PCA in Figure 1. (**A**). Score plot based on PC 1 and PC 2; (**B**). Loading plot based on PC 1 and PC 2; (**C**). Score plot based on PC 1 and PC 3; (**D**). Loading plot based on PC 1 and PC 3; Figure S2: ^1^H NMR spectra of beef extract in 90% H_2_O + 10% D_2_O. A. Unaged samples from heifer (**A**), heifer outlier 1 (**B**) and outlier 2 (**C**); Figure S3: Principal component analysis (PCA) based on the ^1^H NMR spectra (1.1 to 9.0 ppm) of aged beef samples (21 and 28 days); Figure S4: VIP scores of the partial least square regression (PLS-R) in relation to the buckets of ^1^H NMR spectra. The PLS-R is based on samples in various groups: all (**A**), only those from heifers (B), only those from cows (**C**), only unaged and dry-aged samples (**D**), and only unaged and wet-aged samples (**E**); Table S1: Loadings from PCA; Table S2: VIP scores of the PLS-R. Table S3: Generalized linear mixed-effect models for each metabolite.

## Appendix A

**Table A1:** Concentration of metabolites (µmol/g wet meat). Spearman’s rank–order correlation coefficient r_SP_ was calculated for correlation with aging time.

Metabolite	Cattle Type	Aging Type	Concentration in µmol/g Wet Meat over Aging Time					Spearman’s Correlation r_sp_
			0 Days	7 Days	14 Days	21 Days	28 Days	
Amino acids								
Alanine (1.47–1.51 ppm)	H	D W	3.64 ± 0.51 ^a,A^ 3.58 ± 0.56 ^a,A^	3.92 ± 0.75 ^ab,A^ 3.85 ± 0.64 ^ab,A^	4.66 ± 0.83 ^bc,A^ 4.46 ± 0.85 ^bc,A^	5.61 ± 1.39 ^cd,A^ 5.21 ± 0.79 ^cd,A^	6.24 ± 1.11 ^d,A^ 5.85 ± 0.72 ^d,A^	0.78 0.77
	C	D W	3.79 ± 0.32 ^a,A^ 4.02 ± 0.37 ^a,A^	4.16 ± 0.47 ^a,A^ 4.20 ± 0.38 ^a,A^	4.47 ± 0.55 ^a,A^ 4.69 ± 0.51 ^ab,B^	5.45 ± 0.62 ^b,A^ 5.33 ± 0.59 ^bc,A^	6.08 ± 0.73 ^b,A^ 5.79 ± 0.54 ^c,A^	0.80 0.85
	All		3.75 ± 0.48 ^a^	4.02 ± 0.60 ^a^	4.57 ± 0.71 ^b^	5.40 ± 0.93 ^c^	6.00 ± 0.82 ^d^	
Aspartate (2.79–2.80 ppm)	H	D W	0.36 ± 0.21 ^a,A^ 0.37 ± 0.17 ^a,A^	0.47 ± 0.23 ^a,A^ 0.55 ± 0.29 ^ab,B^	0.64 ± 0.26 ^b,A^ 0.61 ± 0.20 ^b,A^	0.69 ± 0.30 ^b,A^ 0.70 ± 0.18 ^bc,A^	0.77 ± 0.23 ^b,A^ 0.85 ± 0.20 ^c,A^	0.58 0.65
	C	D W	0.30 ± 0.08 ^a,A^ 0.31 ± 0.09 ^a,A^	0.35 ± 0.10 ^ab,A^ 0.37 ± 0.10 ^ab,A^	0.46 ± 0.12 ^bc,A^ 0.48 ± 0.12 ^bc,A^	0.54 ± 0.12 ^cd,A^ 0.59 ± 0.14 ^cd,B^	0.73 ± 0.17 ^d,A^ 0.77 ± 0.17 ^d,A^	0.76 0.79
	All		0.33 ± 0.15 ^a^	0.44 ± 0.21 ^b^	0.55 ± 0.20 ^c^	0.64 ± 0.21 ^c^	0.78 ± 0.20 ^d^	
Glutamate (2.32–2.39 ppm)	H	D W	1.21 ± 0.33 ^a,A^ 1.24 ± 0.36 ^a,A^	1.46 ± 0.50 ^ab,A^ 1.57 ± 0.52 ^ab,B^	1.94 ± 0.65 ^bc,A^ 2.09 ± 0.75 ^bc,B^	2.43 ± 0.74 ^cd,A^ 2.62 ± 0.69 ^cd,B^	2.89 ± 0.66 ^d,A^ 3.18 ± 0.64 ^d,B^	0.76 0.81
	C	D W	1.17 ± 0.24 ^a,A^ 1.12 ± 0.17 ^a,A^	1.34 ± 0.26 ^a,A^ 1.42 ± 0.22 ^ab,A^	1.78 ± 0.38 ^ab,A^ 1.92 ± 0.38 ^bc,B^	2.25 ± 0.47 ^bc,A^ 2.46 ± 0.54 ^cd,B^	2.72 ± 0.50 ^c,A^ 2.98 ± 0.50 ^d,B^	0.84 0.87
	All		1.19 ± 0.29 ^a^	1.45 ± 0.41 ^b^	1.94 ± 0.58 ^c^	2.44 ± 0.63 ^d^	2.95 ± 0.60 ^e^	
Glutamine (2.11–2.12 ppm)	H	D W	4.78 ± 1.99 ^a,A^ 5.03 ± 2.33 ^a,A^	5.20 ± 1.57 ^a,A^ 5.22 ± 1.37 ^a,A^	6.43 ± 1.78 ^ab,A^ 6.38 ± 1.73 ^ab,A^	7.61 ± 1.94 ^bc,A^ 7.69 ± 1.82 ^bc,A^	8.64 ± 1.95 ^c,A^ 8.52 ± 1.59 ^c,A^	0.61 0.63
	C	D W	5.04 ± 1.46 ^a,A^ 4.74 ± 1.35 ^a,A^	4.83 ± 0.99 ^a,A^ 5.06 ± 0.83 ^a,A^	6.09 ± 1.10 ^ab,A^ 6.33 ± 1.01 ^ab,A^	7.40 ± 1.31 ^bc,A^ 7.45 ± 1.29 ^bc,A^	8.09 ± 1.12 ^c,A^ 8.32 ± 1.17 ^c,A^	0.69 0.72
	All		4.90 ± 1.83 ^a^	5.09 ± 1.24 ^a^	6.31 ± 1.46 ^b^	7.54 ± 1.62 ^c^	8.40 ± 1.51 ^d^	
Glycine (3.57 ppm)	H	D W	1.19 ± 0.17 ^a,A^ 1.14 ± 0.17 ^a,A^	1.38 ± 0.22 ^ab,A^ 1.38 ± 0.21 ^ab,A^	1.69 ± 0.34 ^bc,A^ 1.62 ± 0.25 ^bc,B^	1.90 ± 0.31 ^cd,A^ 1.83 ± 0.28 ^cd,A^	2.05 ± 0.31 ^d,A^ 2.05 ± 0.29 ^d,A^	0.83 0.83
	C	D W	1.11 ± 0.17 ^a,A^ 1.20 ± 0.20 ^a,B^	1.35 ± 0.26 ^ab,A^ 1.32 ± 0.25 ^a,A^	1.45 ± 0.23 ^bc,A^ 1.50 ± 0.21 ^ab,A^	1.78 ± 0.27 ^cd,A^ 1.75 ± 0.20 ^bc,A^	1.99 ± 0.20 ^d,A^ 1.91 ± 0.21 ^c,A^	0.80 0.80
	All		1.16 ± 0.18 ^a^	1.36 ± 0.23 ^b^	1.57 ± 0.28 ^c^	1.82 ± 0.27 ^d^	2.00 ± 0.26 ^e^	
Isoleucine (1.02–1.03 ppm)	H	D W	0.20 ± 0.06 ^a,A^ 0.20 ± 0.05 ^a,A^	0.34 ± 0.10 ^ab,A^ 0.40 ± 0.12 ^ab,B^	0.55 ± 0.18 ^bc,A^ 0.59 ± 0.19 ^bc,A^	0.76 ± 0.22 ^cd,A^ 0.84 ± 0.20 ^cd,A^	1.02 ± 0.24 ^d,A^ 1.12 ± 0.23 ^d,A^	0.91 0.92
	C	D W	0.20 ± 0.04 ^a,A^ 0.23 ± 0.04 ^a,B^	0.33 ± 0.07 ^ab,A^ 0.36 ± 0.09 ^ab,B^	0.54 ± 0.14 ^bc,A^ 0.60 ± 0.15 ^bc,B^	0.80 ± 0.23 ^cd,A^ 0.90 ± 0.25 ^cd,B^	1.06 ± 0.29 ^d,A^ 1.17 ± 0.28 ^d,B^	0.90 0.92
	All		0.21 ± 0.05 ^a^	0.36 ± 0.10 ^b^	0.57 ± 0.17 ^c^	0.83 ± 0.23 ^d^	1.09 ± 0.26 ^e^	
Leucine (0.97–0.98 ppm)	H	D W	0.33 ± 0.11 ^a,A^ 0.31 ± 0.10 ^a,A^	0.58 ± 0.20 ^ab,A^ 0.70 ± 0.25 ^ab,B^	0.96 ± 0.33 ^bc,A^ 1.01 ± 0.33 ^bc,A^	1.32 ± 0.37 ^cd,A^ 1.42 ± 0.33 ^cd,A^	1.74 ± 0.38 ^d,A^ 1.86 ± 0.34 ^d,A^	0.91 0.92
	C	DW	0.31 ± 0.06 ^a,A^ 0.36 ± 0.06 ^a,B^	0.55 ± 0.14 ^ab,A^ 0.60 ± 0.15 ^ab,B^	0.93 ± 0.27 ^bc,A^ 1.02 ± 0.28 ^bc,B^	1.36 ± 0.40 ^cd,A^ 1.50 ± 0.42 ^cd,B^	1.78 ± 0.47 ^d,A^ 1.95 ± 0.48 ^d,A^	0.90 0.92
	All		0.32 ± 0.09 ^a^	0.61 ± 0.20 ^b^	0.98 ± 0.30 ^c^	1.40 ± 0.38 ^d^	1.83 ± 0.42 ^e^	
Methionine (2.64–2.65 ppm)	H	D W	1.12 ± 0.33 ^a,A^ 1.10 ± 0.28 ^a,A^	1.27 ± 0.28 ^ab,A^ 1.37 ± 0.30 ^ab,B^	1.53 ± 0.27 ^bc,A^ 1.54 ± 0.27 ^bc,A^	1.74 ± 0.32 ^cd,A^ 1.76 ± 0.26 ^cd,A^	1.93 ± 0.32 ^d,A^ 1.94 ± 0.28 ^d,A^	0.74 0.78
	C	D W	1.20 ± 0.13 ^a,A^ 1.22 ± 0.14 ^a,A^	1.39 ± 0.16 ^ab,A^ 1.38 ± 0.16 ^ab,A^	1.59 ± 0.19 ^bc,A^ 1.64 ± 0.20 ^bc,B^	1.88 ± 0.23 ^cd,A^ 1.91 ± 0.26 ^cd,A^	2.12 ± 0.33 ^d,A^ 2.11 ± 0.26 ^d,A^	0.84 0.86
	All		1.16 ± 0.25 ^a^	1.35 ± 0.24 ^b^	1.57 ± 0.24 ^c^	1.82 ± 0.28 ^d^	2.02 ± 0.31 ^e^	
Phenylalanine (7.45–7.47 ppm)	H	D W	0.53 ± 0.04 ^a,A^ 0.52 ± 0.04 ^a,A^	0.64 ± 0.06 ^ab,A^ 0.70 ± 0.09 ^ab,B^	0.82 ± 0.12 ^bc,A^ 0.85 ± 0.13 ^bc,A^	1.00 ± 0.14 ^cd,A^ 1.06 ± 0.14 ^cd,A^	1.19 ± 0.16 ^d,A^ 1.28 ± 0.15 ^d,B^	0.94 0.94
	C	D W	0.51 ± 0.05 ^a,A^ 0.55 ± 0.04 ^a,B^	0.64 ± 0.07 ^ab,A^ 0.68 ± 0.05 ^ab,A^	0.83 ± 0.14 ^bc,A^ 0.88 ± 0.11 ^bc,A^	1.04 ± 0.19 ^cd,A^ 1.14 ± 0.19 ^cd,B^	1.25 ± 0.21 ^d,A^ 1.34 ± 0.22 ^d,B^	0.91 0.94
	All		0.53 ± 0.04 ^a^	0.66 ± 0.07 ^b^	0.84 ± 0.13 ^c^	1.06 ± 0.17 ^d^	1.26 ± 0.19 ^e^	
Tryptophan (7.72–7.75 ppm)	H	D W	0.13 ± 0.01 ^a,A^ 0.13 ± 0.01 ^a,A^	0.15 ± 0.02 ^ab,A^ 0.16 ± 0.02 ^ab,B^	0.19 ± 0.03 ^bc,A^ 0.19 ± 0.03 ^bc,A^	0.22 ± 0.04 ^cd,A^ 0.23 ± 0.03 ^cd,A^	0.25 ± 0.04 ^d,A^ 0.27 ± 0.04 ^d,B^	0.88 0.90
	C	D W	0.13 ± 0.01 ^a,A^ 0.13 ± 0.01 ^a,A^	0.15 ± 0.02 ^ab,A^ 0.16 ± 0.02 ^ab,A^	0.19 ± 0.03 ^bc,A^ 0.20 ± 0.03 ^bc,B^	0.22 ± 0.04 ^cd,A^ 0.24 ± 0.04 ^cd,B^	0.26 ± 0.04 ^d,A^ 0.28 ± 0.04 ^d,B^	0.89 0.91
	All		0.13 ± 0.01 ^a^	0.15 ± 0.02 ^b^	0.19 ± 0.03 ^c^	0.23 ± 0.04 ^d^	0.27 ± 0.04 ^e^	
Tyrosine (7.17–7.22 ppm)	H	D W	0.29 ± 0.06 ^a,A^ 0.29 ± 0.06 ^a,A^	0.43 ± 0.10 ^ab,A^ 0.51 ± 0.13 ^ab,B^	0.64 ± 0.16 ^bc,A^ 0.68 ± 0.18 ^bc,A^	0.82 ± 0.19 ^cd,A^ 0.91 ± 0.18 ^cd,B^	1.04 ± 0.19 ^d,A^ 1.14 ± 0.19 ^d,B^	0.91 0.92
	C	D W	0.30 ± 0.05 ^a,A^ 0.33 ± 0.05 ^a,B^	0.43 ± 0.08 ^ab,A^ 0.48 ± 0.08 ^ab,B^	0.65 ± 0.16 ^bc,A^ 0.71 ± 0.16 ^bc,B^	0.86 ± 0.22 ^cd,A^ 0.96 ± 0.23 ^cd,B^	1.08 ± 0.25 ^d,A^ 1.19 ± 0.24 ^d,B^	0.89 0.92
	All		0.30 ± 0.06 ^a^	0.46 ± 0.11 ^b^	0.67 ± 0.16 ^c^	0.89 ± 0.21 ^d^	1.11 ± 0.22 ^e^	
Valine (1.03–1.06 ppm)	H	D W	0.35 ± 0.08 ^a,A^ 0.33 ± 0.08 ^a,A^	0.55 ± 0.15 ^ab,A^ 0.65 ± 0.18 ^ab,B^	0.89 ± 0.28 ^bc,A^ 0.96 ± 0.30 ^bc,A^	1.24 ± 0.34 ^cd,A^ 1.41 ± 0.32 ^cd,B^	1.67 ± 0.38 ^d,A^ 1.89 ± 0.37 ^d,B^	0.92 0.93
	C	D W	0.36 ± 0.06 ^a,A^ 0.39 ± 0.06 ^a,B^	0.56 ± 0.12 ^ab,A^ 0.61 ± 0.14 ^ab,A^	0.89 ± 0.22 ^bc,A^ 1.00 ± 0.24 ^bc,B^	1.30 ± 0.36 ^cd,A^ 1.50 ± 0.39 ^cd,B^	1.76 ± 0.45 ^d,A^ 1.95 ± 0.45 ^d,B^	0.91 0.93
	All		0.36 ± 0.08 ^a^	0.59 ± 0.15 ^b^	0.93 ± 0.27 ^c^	1.36 ± 0.36 ^d^	1.82 ± 0.42 ^e^	
Nucleotides								
Hypoxanthine (8.22 ppm)	H	D W	1.21 ± 0.32 ^a,A^ 1.32 ± 0.33 ^a,A^	1.96 ± 0.36 ^ab,A^ 2.21 ± 0.50 ^ab,B^	2.72 ± 0.36 ^bc,A^ 2.98 ± 0.43 ^bc,B^	3.41 ± 0.50 ^cd,A^ 3.70 ± 0.44 ^cd,B^	4.15 ± 0.61 ^d,A^ 4.48 ± 0.70 ^d,B^	0.93 0.94
	C	D W	1.12 ± 0.26 ^a,A^ 1.16 ± 0.23 ^a,A^	1.52 ± 0.20 ^ab,A^ 1.81 ± 0.20 ^ab,B^	2.08 ± 0.27 ^bc,A^ 2.43 ± 0.29 ^bc,B^	2.59 ± 0.21 ^cd,A^ 3.07 ± 0.45 ^cd,B^	3.36 ± 0.44 ^d,A^ 3.81 ± 0.77 ^d,B^	0.94 0.96
	All		1.20 ± 0.30 ^a^	1.89 ± 0.42 ^b^	2.57 ± 0.48 ^c^	3.22 ± 0.59 ^d^	3.97 ± 0.76 ^e^	
IMP (6.13–6.16 ppm)	H	D W	3.63 ± 0.29 ^a,A^ 3.45 ± 0.25 ^a,B^	2.88 ± 0.34 ^ab,A^ 2.77 ± 0.34 ^ab,A^	2.16 ± 0.35 ^bc,A^ 2.03 ± 0.41 ^bc,A^	1.67 ± 0.34 ^cd,A^ 1.38 ± 0.32 ^cd,B^	1.17 ± 0.29 ^d,A^ 0.86 ± 0.21 ^d,B^	−0.93 −0.95
	C	D W	3.37 ± 0.35 ^a,A^ 3.32 ± 0.32 ^a,A^	2.92 ± 0.16 ^ab,A^ 2.68 ± 0.21 ^ab,B^	2.43 ± 0.14 ^bc,A^ 2.23 ± 0.15 ^bc,B^	2.07 ± 0.20 ^cd,A^ 1.79 ± 0.24 ^cd,B^	1.65 ± 0.27 ^d,A^ 1.39 ± 0.24 ^d,B^	−0.93 −0.94
	All		3.43 ± 0.33 ^a^	2.81 ± 0.29 ^b^	2.20 ± 0.32 ^c^	1.71 ± 0.38 ^d^	1.25 ± 0.38 ^e^	
Inosine (6.08–6.12 ppm)	H	D W	0.85 ± 0.10 ^a,A^ 0.86 ± 0.10 ^a,A^	1.26 ± 0.17 ^ab,A^ 1.19 ± 0.08 ^a,A^	1.52 ± 0.11 ^bc,A^ 1.44 ± 0.08 ^b,B^	1.69 ± 0.15 ^c,A^ 1.48 ± 0.16 ^b,B^	1.66 ± 0.14 ^c,A^ 1.49 ± 0.08 ^b,B^	0.88 0.87
	C	D W	0.79 ± 0.11 ^a,A^ 0.83 ± 0.13 ^a,A^	1.14 ± 0.19 ^ab,A^ 1.03 ± 0.16 ^ab,B^	1.31 ± 0.19 ^bc,A^ 1.17 ± 0.21 ^bc,B^	1.48 ± 0.24 ^c,A^ 1.21 ± 0.25 ^c,B^	1.44 ± 0.28 ^c,A^ 1.24 ± 0.25 ^c,B^	0.80 0.68
	All		0.84 ± 0.11 ^a^	1.16 ± 0.17 ^b^	1.37 ± 0.20 ^c^	1.47 ± 0.26 ^c^	1.46 ± 0.25 ^c^	
Acids								
Acetic acid (1.92–1.93 ppm)	H	D W	0.39 ± 0.10 ^a,A^ 0.40 ± 0.09 ^a,A^	0.49 ± 0.12 ^ab,A^ 0.49 ± 0.11 ^ab,A^	0.61 ± 0.14 ^bc,A^ 0.58 ± 0.13 ^bc,A^	0.74 ± 0.19 ^cd,A^ 0.70 ± 0.12 ^cd,A^	0.88 ± 0.17 ^d,A^ 0.83 ± 0.12 ^d,A^	0.83 0.85
	C	D W	0.47 ± 0.11 ^a,A^ 0.49 ± 0.06 ^a,A^	0.59 ± 0.08 ^ab,A^ 0.59 ± 0.06 ^ab,A^	0.66 ± 0.09 ^bc,A^ 0.68 ± 0.10 ^bc,A^	0.77 ± 0.08 ^cd,A^ 0.80 ± 0.12 ^cd,A^	0.91 ± 0.15 ^d,A^ 1.03 ± 0.22 ^d,A^	0.86 0.87
	All		0.43 ± 0.10 ^a^	0.54 ± 0.11 ^b^	0.63 ± 0.12 ^c^	0.75 ± 0.14 ^d^	0.91 ± 0.18 ^e^	
Fumaric Acid (6.52–6.53 ppm)	H	D W	0.19 ± 0.09 ^a,A^ 0.18 ± 0.10 ^a,A^	0.24 ± 0.08 ^a,A^ 0.26 ± 0.12 ^ab,A^	0.36 ± 0.15 ^b,A^ 0.30 ± 0.10 ^bc,A^	0.42 ± 0.20 ^b,A^ 0.34 ± 0.09 ^bc,A^	0.45 ± 0.12 ^b,A^ 0.44 ± 0.15 ^c,A^	0.68 0.64
	C	D W	0.22 ± 0.08 ^a,A^ 0.26 ± 0.06 ^a,A^	0.29 ± 0.11 ^ab,A^ 0.29 ± 0.09 ^ab,A^	0.35 ± 0.08 ^b,A^ 0.39 ± 0.09 ^c,A^	0.31 ± 0.09 ^ab,A^ 0.34 ± 0.10 ^abc,A^	0.42 ± 0.15 ^b,A^ 0.37 ± 0.12 ^bc,A^	0.50 0.51
	All		0.21 ± 0.09 ^a^	0.27 ± 0.10 ^b^	0.35 ± 0.11 ^c^	0.35 ± 0.14 ^c^	0.42 ± 0.14 ^d^	
Lactic Acid (1.30–1.37 ppm)	H	D W	70.22 ± 5.02 ^a,A^ 66.64 ± 8.95 ^a,A^	72.84 ± 5.94 ^ab,A^ 70.39 ± 5.28 ^a,A^	76.92 ± 8.19 ^abc,A^ 69.15 ± 5.04 ^a,B^	81.32 ± 8.77 ^bc,A^ 70.39 ± 4.57 ^a,B^	83.27 ± 9.49 ^c,A^ 70.71 ± 4.40 ^a,B^	0.62 0.08
	C	D W	70.02 ± 4.00 ^a,A^ 70.89 ± 4.12 ^a,A^	75.51 ± 4.60 ^ab,A^ 72.29 ± 3.91 ^ab,A^	78.07 ± 4.64 ^bc,A^ 74.85 ± 2.11 ^bc,A^	87.89 ± 8.84 ^c,A^ 76.01 ± 4.07 ^c,B^	90.45 ± 11.62 ^c,A^ 77.37 ± 3.16 ^c,B^	0.79 0.66
	All		69.37 ± 6.15 ^a^	72.68 ± 5.30 ^b^	74.63 ± 6.50 ^bc^	78.70 ± 9.43 ^cd^	80.22 ± 10.71 ^d^	
Succinic Acid (2.41–2.42 ppm)	H	D W	0.37 ± 0.12 ^a,A^ 0.36 ± 0.11 ^a,A^	0.62 ± 0.36 ^ab,A^ 0.51 ± 0.21 ^ab,A^	0.68 ± 0.29 ^bc,A^ 0.70 ± 0.37 ^bc,A^	0.86 ± 0.34 ^c,A^ 1.00 ± 0.52 ^c,A^	0.97 ± 0.60 ^c,A^ 0.86 ± 0.31 ^c,A^	0.69 0.71
	C	D W	0.59 ± 0.19 ^a,A^ 0.82 ± 0.21 ^a,A^	0.96 ± 0.22 ^b,A^ 1.02 ± 0.20 ^ab,A^	0.98 ± 0.23 ^b,A^ 1.00 ± 0.17 ^ab,A^	1.14 ± 0.28 ^b,A^ 1.11 ± 0.24 ^b,A^	1.05 ± 0.31 ^b,A^ 1.13 ± 0.25 ^b,B^	0.49 0.57
	All		0.52 ± 0.25 ^a^	0.76 ± 0.34 ^b^	0.83 ± 0.31 ^bc^	1.02 ± 0.38 ^d^	1.00 ± 0.40 ^cd^	
Sugars								
α Glucose (5.25–5.27 ppm)	H	D W	2.12 ± 0.39 ^a,A^ 1.88 ± 0.53 ^a,A^	2.67 ± 0.49 ^ab,A^ 2.90 ± 0.56 ^ab,A^	3.89 ± 0.69 ^bc,A^ 3.90 ± 0.59 ^bc,A^	4.48 ± 0.60 ^c,A^ 4.61 ± 0.85 ^c,A^	4.70 ± 0.73 ^c,A^ 4.71 ± 0.70 ^c,A^	0.88 0.88
	C	D W	2.10 ± 0.50 ^a,A^ 2.19 ± 0.37 ^a,A^	2.90 ± 0.45 ^ab,A^ 2.87 ± 0.38 ^ab,A^	3.59 ± 0.84 ^bc,A^ 3.69 ± 0.46 ^bc,A^	4.53 ± 0.70 ^cd,A^ 4.42 ± 0.73 ^c,A^	5.07 ± 0.88 ^d,A^ 4.58 ± 0.77 ^c,A^	0.90 0.89
	All		2.07 ± 0.46 ^a^	2.83 ± 0.48 ^b^	3.77 ± 0.66 ^c^	4.51 ± 0.72 ^d^	4.76 ± 0.78 ^d^	
β Glucose (4.64–4.67 ppm)	H	D W	3.61 ± 0.79 ^a,A^ 3.47 ± 1.68 ^a,A^	4.43 ± 0.96 ^a,A^ 4.78 ± 1.11 ^ab,A^	6.00 ± 1.01 ^b,A^ 6.04 ± 0.95 ^bc,A^	6.63 ± 0.88 ^b,A^ 6.69 ± 1.16 ^c,A^	6.49 ± 0.96 ^b,A^ 6.51 ± 1.02 ^c,A^	0.80 0.76
	C	D W	3.45 ± 1.09 ^a,A^ 3.71 ± 0.78 ^a,A^	4.59 ± 0.97 ^ab,A^ 4.53 ± 0.77 ^ab,A^	5.68 ± 2.33 ^bc,A^ 5.50 ± 0.92 ^bc,A^	6.66 ± 1.23 ^cd,A^ 6.38 ± 1.24 ^c,A^	6.92 ± 1.26 ^d,A^ 6.32 ± 1.18 ^c,A^	0.80 0.78
	All		3.56 ± 1.15 ^a^	4.58 ± 0.96 ^b^	5.82 ± 1.41 ^c^	6.59 ± 1.12 ^d^	6.55 ± 1.11 ^d^	
Glucose-6-phosphate (5.22–5.24 ppm)	H	D W	1.53 ± 0.44 ^a,A^ 1.35 ± 0.58 ^a,A^	1.75 ± 0.47 ^ab,A^ 1.87 ± 0.45 ^abc,A^	2.19 ± 0.40 ^c,A^ 2.16 ± 0.39 ^b,A^	2.12 ± 0.41 ^bc,A^ 2.07 ± 0.43 ^bc,A^	1.81 ± 0.35 ^ab,A^ 1.75 ± 0.31 ^ac,A^	0.40 0.34
	C	D W	1.47 ± 0.54 ^a,A^ 1.61 ± 0.39 ^a,A^	1.75 ± 0.53 ^ab,A^ 1.70 ± 0.35 ^a,A^	1.93 ± 0.65 ^ab,A^ 1.89 ± 0.42 ^a,A^	2.08 ± 0.46 ^b,A^ 1.97 ± 0.50 ^a,A^	1.94 ± 0.40 ^b,A^ 1.76 ± 0.41 ^a,A^	0.35 0.23
	All		1.49 ± 0.50 ^a^	1.77 ± 0.46 ^b^	2.05 ± 0.48 ^c^	2.06 ± 0.45 ^c^	1.81 ± 0.37 ^d^	
Further compounds								
Anserine (7.08–7.10 ppm)	H	D W	1.61 ± 0.66 ^a,A^ 1.72 ± 0.70 ^a,A^	1.96 ± 0.68 ^ab,A^ 2.03 ± 0.76 ^ab,A^	2.40 ± 0.91 ^bc,A^ 2.39 ± 0.89 ^bc,A^	2.65 ± 0.95 ^c,A^ 2.63 ± 1.00 ^c,A^	2.89 ± 1.10 ^c,A^ 2.59 ± 0.99 ^c,A^	0.51 0.45
	C	D W	2.12 ± 0.46 ^a,A^ 2.16 ± 0.38 ^a,A^	2.24 ± 0.51 ^ab,A^ 2.18 ± 0.47 ^a,A^	2.58 ± 0.46 ^abc,A^ 2.56 ± 0.44 ^ab,A^	3.03 ± 0.53 ^c,A^ 2.57 ± 0.51 ^ab,B^	2.76 ± 0.88 ^bc,A^ 2.93 ± 0.48 ^b,A^	0.44 0.51
	All		1.89 ± 0.62 ^a^	2.09 ± 0.63 ^a^	2.48 ± 0.72 ^b^	2.71 ± 0.81 ^b^	2.79 ± 0.91 ^b^	
Betaine (3.27–3.28 ppm)	H	D W	4.50 ± 0.81 ^a,A^ 4.33 ± 0.87 ^a,A^	5.38 ± 0.97 ^ab,A^ 5.60 ± 1.16 ^ab,A^	6.64 ± 0.86 ^bc,A^ 6.54 ± 0.91 ^bc,A^	7.37 ± 1.30 ^c,A^ 7.23 ± 1.02 ^cd,A^	7.95 ± 1.37 ^c,A^ 7.66 ± 0.81 ^d,A^	0.82 0.81
	C	D W	4.47 ± 0.66 ^a,A^ 4.56 ± 0.67 ^a,A^	5.16 ± 0.79 ^ab,A^ 5.19 ± 0.64 ^ab,A^	6.06 ± 0.94 ^bc,A^ 6.11 ± 0.80 ^bc,A^	7.12 ± 1.07 ^cd,A^ 6.87 ± 0.98 ^cd,B^	8.12 ± 1.29 ^d,A^ 7.56 ± 1.08 ^d,A^	0.83 0.84
	All		4.46 ± 0.76 ^a^	5.34 ± 0.93 ^b^	6.35 ± 0.91 ^c^	7.16 ± 1.11 ^d^	7.82 ± 1.16 ^e^	
Carnitine (3.22–3.23 ppm)	H	D W	4.57 ± 0.55 ^ab,A^ 4.45 ± 0.71 ^a,A^	4.19 ± 0.42 ^a,A^ 4.20 ± 0.42 ^ab,A^	4.31 ± 0.33 ^ab,A^ 4.10 ± 0.37 ^b,A^	4.66 ± 0.61 ^ab,A^ 4.23 ± 0.35 ^ab,B^	4.65 ± 0.47 ^b,A^ 4.11 ± 0.42 ^b,B^	0.09 −0.31
	C	D W	2.19 ± 0.27 ^a,A^ 2.21 ± 0.23 ^a,A^	2.29 ± 0.31 ^a,A^ 2.20 ± 0.20 ^a,A^	2.29 ± 0.23 ^a,A^ 2.26 ± 0.20 ^a,A^	2.61 ± 0.36 ^b,A^ 2.37 ± 0.24 ^ab,B^	2.65 ± 0.44 ^b,A^ 2.41 ± 0.23 ^b,A^	0.45 0.31
	All		3.43 ± 1.26 ^a^	3.28 ± 1.04 ^a^	3.30 ± 1.01 ^a^	3.53 ± 1.08 ^a^	3.52 ± 1.03 ^a^	
Carnosine (7.10–7.14 ppm)	H	D W	16.12 ± 5.86 ^a,A^ 15.92 ± 5.76 ^a,A^	16.77 ± 5.79 ^a,A^ 17.56 ± 5.93 ^b,B^	18.02 ± 6.30 ^ab,A^ 17.90 ± 6.16 ^b,A^	18.93 ± 6.53 ^b,A^ 18.24 ± 6.26 ^b,A^	18.76 ± 6.24 ^b,A^ 18.10 ± 6.01 ^b,A^	0.37 0.30
	C	D W	18.56 ± 2.46 ^a,A^ 18.70 ± 2.74 ^a,A^	19.53 ± 2.52 ^ab,A^ 19.18 ± 2.03 ^a,A^	20.35 ± 1.96 ^abc,A^ 20.35 ± 1.95 ^ab,A^	22.39 ± 1.68 ^c,A^ 20.87 ± 2.06 ^bc,B^	21.57 ± 3.60 ^bc,A^ 22.03 ± 1.85 ^c,A^	0.46 0.47
	All		17.24 ± 4.73 ^a^	18.19 ± 4.64 ^a^	19.08 ± 4.84 ^b^	20.01 ± 5.06 ^b^	20.00 ± 5.12 ^b^	
Creatine (3.92–3.94 ppm)	H	D W	32.40 ± 2.45 ^a,A^ 30.92 ± 1.09 ^a,B^	32.64 ± 3.36 ^a,A^ 32.31 ± 2.51 ^a,A^	34.23 ± 4.35 ^ab,A^ 32.59 ± 2.72 ^a,B^	35.52 ± 3.07 ^b,A^ 32.54 ± 2.20 ^a,B^	34.66 ± 2.88 ^ab,A^ 31.76 ± 2.40 ^a,B^	0.48 0.05
	C	D W	30.21 ± 1.61 ^a,A^ 30.18 ± 1.73 ^a,A^	31.52 ± 1.65 ^a,A^ 30.37 ± 2.10 ^a,B^	31.84 ± 1.84 ^ab,A^ 31.40 ± 1.38 ^ab,A^	34.74 ± 3.01 ^b,A^ 31.88 ± 1.68 ^b,B^	34.71 ± 3.01 ^b,A^ 31.32 ± 1.49 ^ab,B^	0.64 0.31
	All		30.97 ± 1.99 ^a^	31.76 ± 2.64 ^ab^	32.58 ± 3.04 ^bc^	33.69 ± 2.95 ^d^	33.12 ± 2.95 ^cd^	
Creatinine (4.06–4.07 ppm)	H	D W	1.23 ± 0.31 ^a,A^ 1.16 ± 0.29 ^a,A^	1.69 ± 0.42 ^ab,A^ 1.91 ± 0.50 ^b,A^	2.07 ± 0.42 ^bc,A^ 2.06 ± 0.40 ^b,A^	2.16 ± 0.39 ^bc,A^ 2.05 ± 0.30 ^b,A^	2.41 ± 0.40 ^c,A^ 2.20 ± 0.22 ^b,A^	0.79 0.74
	C	D W	1.39 ± 0.44 ^a,A^ 1.40 ± 0.35 ^a,A^	1.67 ± 0.40 ^ab,A^ 1.78 ± 0.47 ^ab,A^	2.12 ± 0.44 ^bc,A^ 2.05 ± 0.27 ^b,A^	2.17 ± 0.29 ^c,A^ 2.09 ± 0.24 ^bc,A^	2.44 ± 0.39 ^c,A^ 2.38 ± 0.28 ^c,A^	0.73 0.72
	All		1.29 ± 0.36 ^a^	1.76 ± 0.45 ^b^	2.08 ± 0.39 ^c^	2.12 ± 0.31 ^c^	2.35 ± 0.34 ^d^	
Niacinamide (8.71–8.73 ppm)	H	D W	0.42 ± 0.03 ^a,A^ 0.40 ± 0.04 ^a,A^	0.43 ± 0.04 ^a,A^ 0.48 ± 0.03 ^ab,B^	0.50 ± 0.05 ^b,A^ 0.50 ± 0.03 ^bc,A^	0.51 ± 0.06 ^b,A^ 0.53 ± 0.03 ^c,A^	0.54 ± 0.06 ^b,A^ 0.53 ± 0.03 ^c,A^	0.74 0.86
	C	D W	0.36 ± 0.04 ^a,A^ 0.37 ± 0.03 ^a,A^	0.41 ± 0.05 ^ab,A^ 0.41 ± 0.04 ^ab,A^	0.47 ± 0.05 ^bc,A^ 0.50 ± 0.03 ^bc,B^	0.52 ± 0.07 ^c,A^ 0.54 ± 0.04 ^c,A^	0.55 ± 0.08 ^c,A^ 0.54 ± 0.04 ^c,A^	0.81 0.87
	All		0.39 ± 0.04 ^a^	0.43 ± 0.05 ^b^	0.49 ± 0.04 ^c^	0.53 ± 0.05 ^d^	0.54 ± 0.05 ^d^	
O-Acetyl-L-carnitine (5.57–5.65 ppm)	H	D W	1.50 ± 0.36 ^a,A^ 1.62 ± 0.50 ^a,A^	1.95 ± 0.25 ^b,A^ 1.79 ± 0.26 ^ab,B^	2.04 ± 0.22 ^b,A^ 1.91 ± 0.19 ^b,A^	2.04 ± 0.28 ^b,A^ 1.84 ± 0.20 ^ab,B^	1.97 ± 0.28 ^ab,A^ 1.81 ± 0.24 ^ab,B^	0.49 0.34
	C	D W	0.93 ± 0.16 ^a,A^ 0.98 ± 0.22 ^a,A^	0.98 ± 0.17 ^a,A^ 0.97 ± 0.19 ^a,A^	0.99 ± 0.28 ^a,A^ 0.94 ± 0.15 ^ab,A^	0.91 ± 0.13 ^a,A^ 0.86 ± 0.13 ^bc,A^	0.89 ± 0.14 ^a,A^ 0.82 ± 0.12 ^c,B^	−0.15 −0.31
	All		1.28 ± 0.46 ^a^	1.45 ± 0.50 ^ab^	1.50 ± 0.55 ^b^	1.45 ± 0.57 ^ab^	1.41 ± 0.56 ^ab^	

H—heifer; C—cow; D—dry-aged; W—wet-aged; ^a–e^ different letters in the same row indicate a significant difference (*p* < 0.05) between aging time; ^A–B^ different letters in the same column indicate a significant difference (*p* < 0.05) between aging types; r_sp_ Spearman’s rank–order correlation coefficient.
