# Supplementary material for: Variations in the Metabolome of Unaged and Aged Beef from Black-and-White Cows and Heifers by 1H NMR Spectroscopy
Source: Foods. 2023 Feb 13;12(4):785. doi: 10.3390/foods12040785 (PMC9956905; doi:10.3390/foods12040785)
Supplement: Supplementary file 1 [file foods-12-00785-s001.zip › foods-2145957-supplementary.pdf]

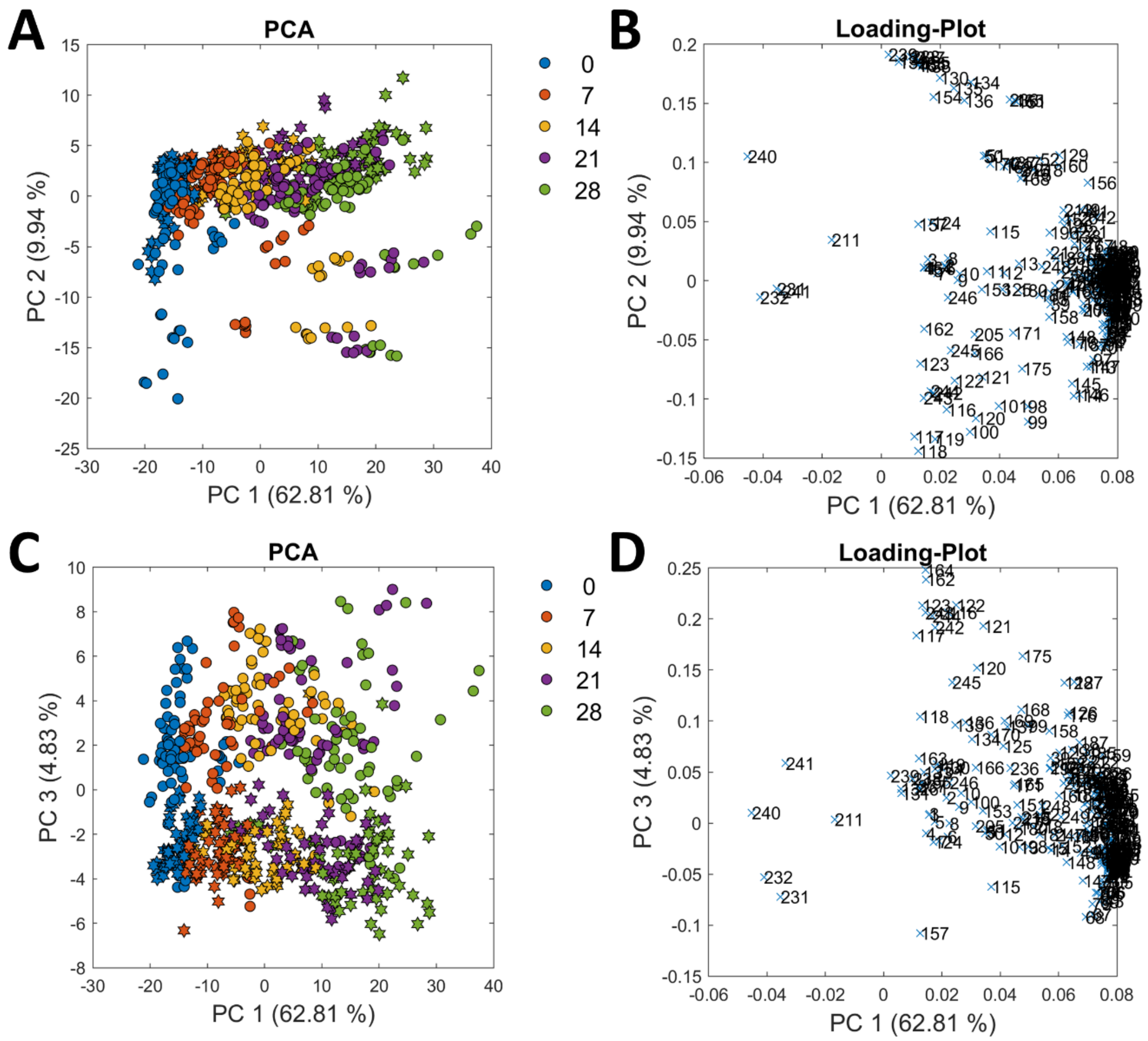

Figure S1. Further display options of the calculated PC in Figure 1.(**A**). Score plot based on PC 1 and PC 2; (**B**). Loading plot based on PC 1 and PC 2; (**C**). Score plot based on PC 1 and PC 3; (**D**). Loading plot based on PC 1 and PC 3.

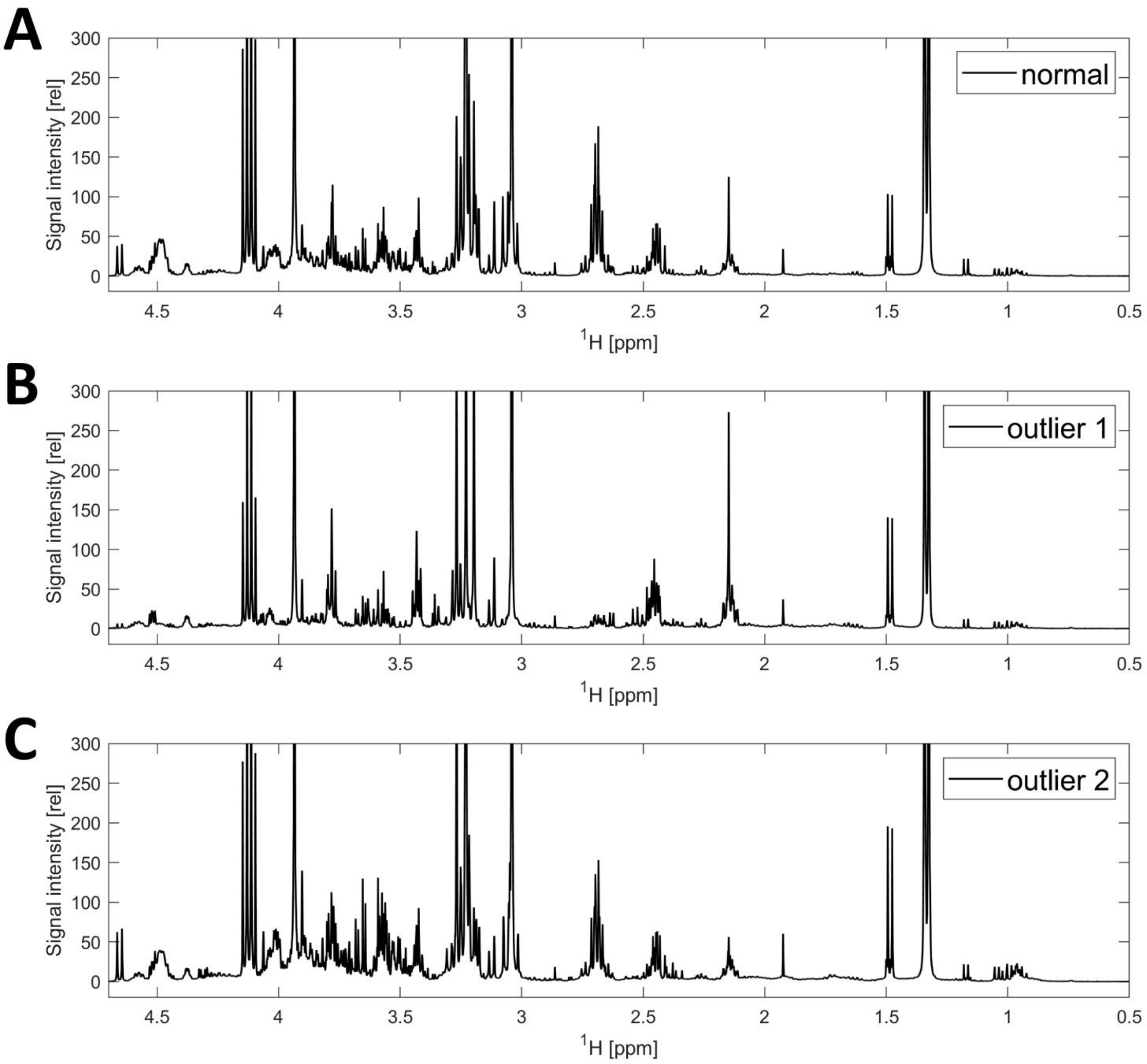

Figure S2. ^{1}H NMR spectra of beef extract in 90 % H_2O + 10 % D_2O. A. Unaged samples from heifer (**A**), heifer outlier 1 (**B**) and outlier 2 (**C**).

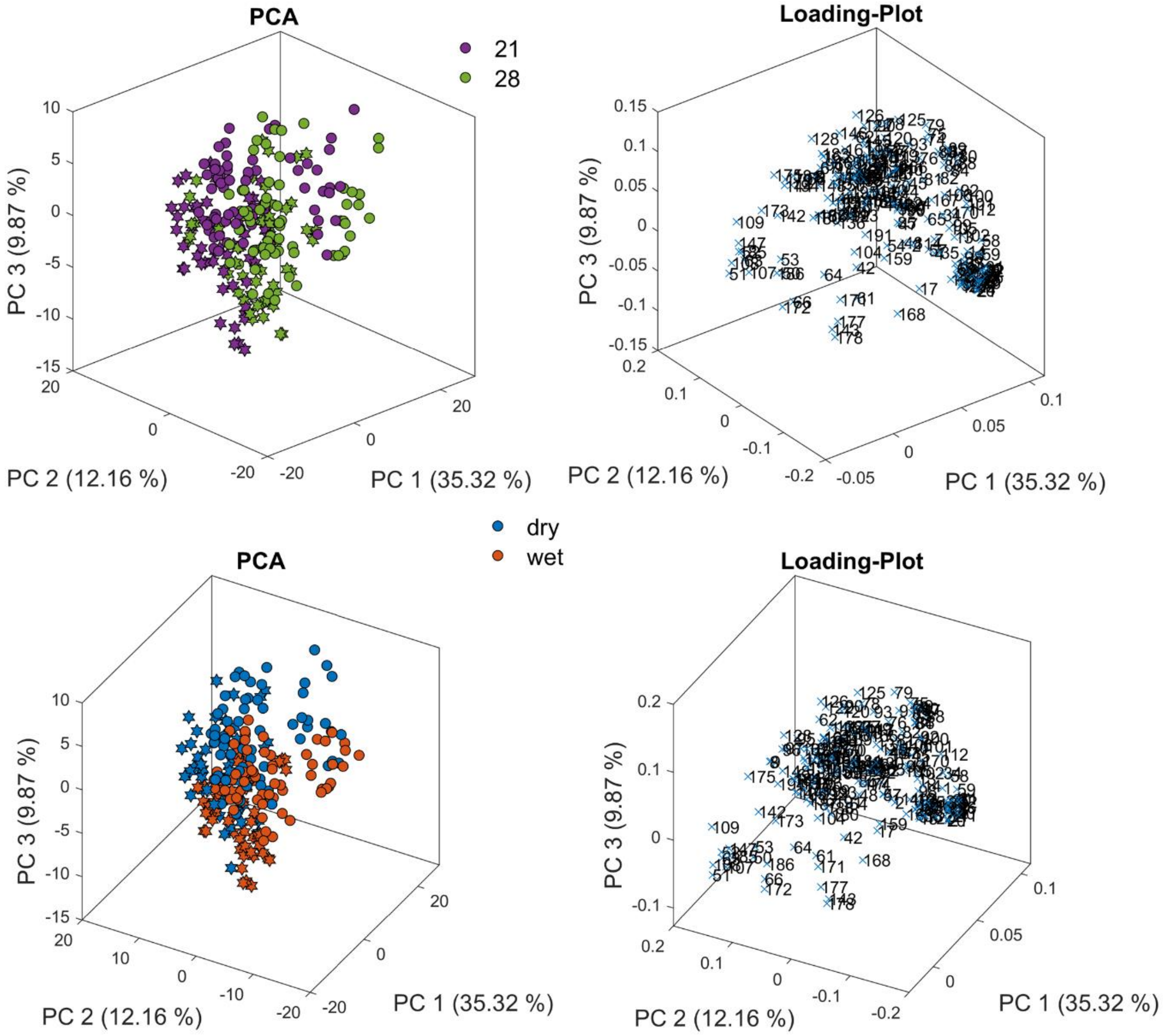

Figure S3. Principal component analysis (PCA) based on the ^{1}H NMR spectra (1.1 to 9.0 ppm) of aged beef samples (21 and 28 days).

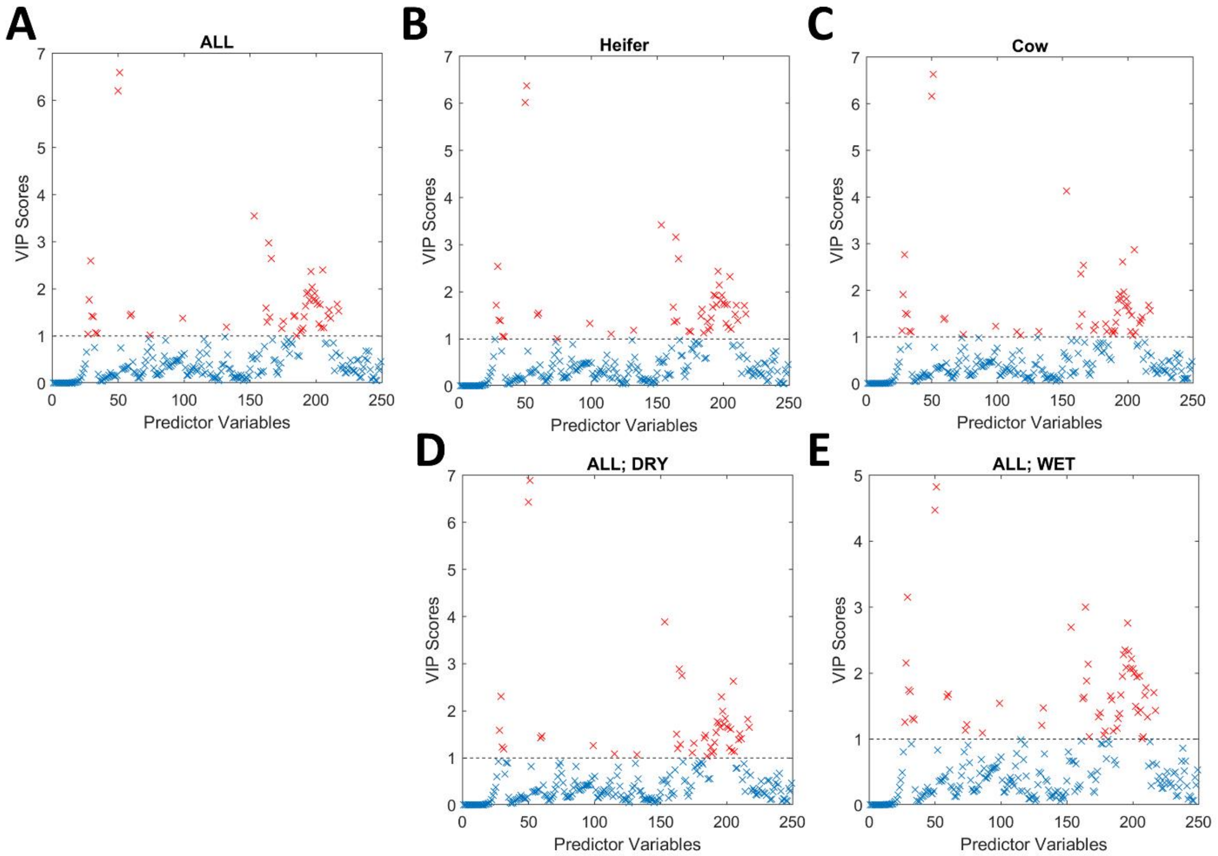

Figure S4. VIP scores of the partial least square regression (PLS-R) in relation to the buckets of ^{1}H NMR spectra. The PLS-R is based on samples in various groups: all (**A**), only those from heifers (**B**), only those from cows (**C**), only unaged and dry-aged samples (**D**), and only unaged and wet-aged samples (E).

Table S1. Loadings from PCA.

Bucket-numbe r	ppm	PC 1	PC 2	PC 3	Metabolite
**1**	0.5000	0.01545	0.01105	0.00795	
**2**	0.5167	0.02157	0.01288	0.02484	
**3**	0.5335	0.01574	0.01788	0.00900	
**4**	0.5502	0.01457	0.01082	-0.01008	
**5**	0.5670	0.01759	0.00891	0.00126	
**6**	0.5837	0.02196	0.01080	-0.01320	
**7**	0.6005	0.01893	0.00606	-0.01791	
**8**	0.6172	0.02258	0.01901	-0.00025	
**9**	0.6340	0.02589	0.00052	0.01559	
**10**	0.6507	0.02661	0.00590	0.02929	
**11**	0.6675	0.03579	0.00790	-0.00683	
**12**	0.6842	0.04139	0.00661	-0.01090	
**13**	0.7010	0.04660	0.01458	-0.02367	
**14**	0.7177	0.05754	-0.01072	-0.02649	
**15**	0.7345	0.05642	-0.01498	-0.02441	
**16**	0.7512	0.06449	-0.00781	-0.01319	
**17**	0.7680	0.07215	-0.00696	-0.02622	
**18**	0.7847	0.07456	-0.00165	-0.03883	
**19**	0.8014	0.07689	0.00261	-0.04014	
**20**	0.8182	0.07698	-0.00330	-0.04058	
**21**	0.8349	0.07683	-0.00139	-0.04423	
**22**	0.8517	0.07675	-0.00188	-0.04536	
**23**	0.8684	0.07687	-0.00453	-0.04287	
**24**	0.8852	0.07702	-0.00255	-0.04408	
**25**	0.9019	0.07732	0.00168	-0.04944	Isoleucine
**26**	0.9187	0.07802	-0.00122	-0.04345	Isoleucine
**27**	0.9354	0.07802	-0.00127	-0.04282	Isoleucine, Leucine
**28**	0.9522	0.07815	-0.00239	-0.03803	Isoleucine, Leucine
**29**	0.9689	0.07797	-0.00077	-0.03780	Leucine, Valine
**30**	0.9857	0.07753	-0.00009	-0.03965	Isoleucine, Valine
**31**	1.0024	0.07704	-0.00093	-0.04148	Isoleucine
**32**	1.0192	0.07748	-0.00043	-0.04048	Isoleucine, Valine
**33**	1.0359	0.07670	-0.00118	-0.04686	Valine
**34**	1.0527	0.07666	-0.00196	-0.04736	Valine
**35**	1.0694	0.07797	-0.00050	-0.03311	
**36**	1.0861	0.07644	-0.00248	0.02691	
**37**	1.1029	0.07581	-0.02429	0.02936	
**38**	1.1196	0.07772	-0.00737	-0.01461	
**39**	1.1364	0.05728	-0.01997	0.06377	
**40**	1.1531	0.06283	-0.00306	0.04189	
**41**	1.1699	0.07032	0.05862	-0.01446	
**42**	1.1866	0.07276	0.05356	-0.03114	Isoleucine
**43**	1.2034	0.07579	0.01135	-0.07638	Isoleucine
**44**	1.2201	0.07781	0.00547	-0.05524	Isoleucine
**45**	1.2369	0.07859	-0.00312	-0.02944	Isoleucine
**46**	1.2536	0.07865	-0.00338	-0.02892	Isoleucine
**47**	1.2704	0.07845	0.00821	-0.03085	Isoleucine
**48**	1.2871	0.07689	0.02931	-0.02658	Isoleucine
**49**	1.3039	0.06746	0.06022	-0.02919	Isoleucine, Lactic acid
**50**	1.3206	0.03465	0.10536	-0.00983	Isoleucine, Lactic acid

Bucket-number	ppm	PC 1	PC 2	PC 3	Metabolite
**51**	1.3373	0.03541	0.10599	-0.00803	Lactic acid
**52**	1.3541	0.05364	0.10287	0.00857	Lactic acid
**53**	1.3708	0.07725	0.02442	-0.02594	
**54**	1.3876	0.07834	0.00362	-0.03216	
**55**	1.4043	0.07825	-0.00703	-0.03458	Isoleucine
**56**	1.4211	0.07826	-0.01447	-0.03692	Isoleucine
**57**	1.4378	0.07764	-0.02616	-0.04250	Isoleucine
**58**	1.4546	0.07714	-0.03489	-0.04215	Isoleucine
**59**	1.4713	0.07079	-0.01007	-0.01173	Isoleucine
**60**	1.4881	0.07058	-0.00229	-0.00887	Isoleucine, Alanine
**61**	1.5048	0.07695	0.01629	-0.00223	Isoleucine, Alanine
**62**	1.5216	0.07759	-0.00210	-0.00432	Isoleucine, Alanine
**63**	1.5383	0.07829	0.00486	-0.03697	Isoleucine
**64**	1.5551	0.07770	0.00778	-0.04808	
**65**	1.5718	0.07791	0.00978	-0.04887	
**66**	1.5886	0.07640	0.01356	-0.06741	
**67**	1.6053	0.07163	0.01814	-0.08942	
**68**	1.6220	0.06925	0.01814	-0.09201	Leucine
**69**	1.6388	0.07413	0.00111	-0.07710	Leucine
**70**	1.6555	0.07329	-0.05641	-0.06806	Leucine
**71**	1.6723	0.07436	-0.05406	-0.05655	Leucine
**72**	1.6890	0.07747	-0.02492	-0.04478	Leucine
**73**	1.7058	0.07745	-0.02369	-0.04309	Leucine
**74**	1.7225	0.07751	-0.02265	-0.03917	Leucine
**75**	1.7393	0.07733	-0.02681	-0.03546	Leucine
**76**	1.7560	0.07747	-0.02528	-0.03651	Leucine
**77**	1.7728	0.07762	-0.01889	-0.04198	Leucine
**78**	1.7895	0.07458	-0.01259	-0.06265	Leucine
**79**	1.8063	0.07151	-0.01635	-0.07908	
**80**	1.8230	0.07451	-0.02182	-0.06950	
**81**	1.8398	0.07699	-0.02977	-0.05187	
**82**	1.8565	0.07710	-0.03925	-0.04028	
**83**	1.8733	0.07647	-0.04780	-0.03831	
**84**	1.8900	0.07685	-0.04378	-0.03153	
**85**	1.9067	0.07736	-0.03538	-0.02537	Acetic acid
**86**	1.9235	0.07456	0.00083	-0.03524	Isoleucine
**87**	1.9402	0.07785	-0.02954	-0.01552	Isoleucine
**88**	1.9570	0.07807	-0.02793	-0.01979	Isoleucine
**89**	1.9737	0.07845	-0.01988	-0.02901	Isoleucine
**90**	1.9905	0.07837	-0.02008	-0.03362	Isoleucine
**91**	2.0072	0.07793	-0.02554	-0.03383	Isoleucine
**92**	2.0240	0.07775	-0.03136	-0.02961	Isoleucine, Glutamate
**93**	2.0407	0.07735	-0.03693	-0.02885	Glutamate, Glutamine
**94**	2.0575	0.07560	-0.05371	-0.03392	Glutamate, O-Acetyl-L-carnitine, Glutamine
**95**	2.0742	0.07609	-0.04980	-0.03681	Glutamate, Methionine, O-Acetyl-L-carnitine, Glutamine
**96**	2.0910	0.07660	-0.04307	-0.03641	Glutamate, Methionine, O-Acetyl-L-carnitine, Glutamine
**97**	2.1077	0.07165	-0.06661	-0.02912	Glutamate, Methionine, O-Acetyl-L-carnitine, Glutamine
**98**	2.1245	0.04960	-0.10629	-0.02303	Glutamate, Methionine, O-Acetyl-L-carnitine, Glutamine

Bucket-numbe r	ppm	PC 1	PC 2	PC 3	Metabolite
**99**	2.1412	0.04961	-0.11933	0.09530	Glutamate, Methionine, O-Acetyl-L-carnitine, Glutamine
**100**	2.1580	0.02997	-0.12784	0.02067	Glutamate, Methionine, O-Acetyl-L-carnitine, Glutamine
**101**	2.1747	0.03974	-0.10592	-0.02324	Glutamate, Methionine, O-Acetyl-L-carnitine, Glutamine
**102**	2.1914	0.07488	-0.03674	-0.02706	Methionine, O-Acetyl-L-carnitine, Glutamine
**103**	2.2082	0.07839	-0.01484	-0.02493	Valine, Methionine, O-Acetyl-L-carnitine, Glutamine
**104**	2.2249	0.07835	-0.00478	-0.03318	Valine, Methionine, Glutamine
**105**	2.2417	0.07562	0.01270	-0.05765	Valine, Methionine, Glutamine
**106**	2.2584	0.07270	0.01475	-0.06812	Valine, Methionine, Glutamine
**107**	2.2752	0.07748	-0.00001	-0.01539	Valine, Glutamine
**108**	2.2919	0.07865	-0.01476	-0.01965	Valine, Glutamine
**109**	2.3087	0.07842	-0.02034	-0.01603	Valine, Glutamine
**110**	2.3254	0.07765	-0.03154	-0.03435	Valine, Glutamate
**111**	2.3422	0.07574	-0.03641	-0.02441	Glutamate, Succinic acid
**112**	2.3589	0.07483	-0.04206	-0.02419	Glutamate, Succinic acid
**113**	2.3757	0.06955	-0.07280	-0.00532	Glutamate, Carnitine, Glutamine, Succinic acid
**114**	2.3924	0.06509	-0.09740	0.04466	Carnitine, Glutamine, Succinic acid
**115**	2.4092	0.03689	0.04164	-0.06234	Carnitine, Glutamine, Succinic acid
**116**	2.4259	0.02211	-0.10901	0.20639	Carnitine, Glutamine, Succinic acid
**117**	2.4427	0.01128	-0.13187	0.18386	Carnitine, Glutamine, Succinic acid
**118**	2.4594	0.01249	-0.14422	0.10437	Carnitine, O-Acetyl-L-carnitine, Glutamine, Succinic acid
**119**	2.4761	0.01818	-0.13395	0.05739	Carnitine, O-Acetyl-L-carnitine, Glutamine, Succinic acid
**120**	2.4929	0.03204	-0.11642	0.15214	Carnitine, O-Acetyl-L-carnitine, Glutamine, Succinic acid
**121**	2.5096	0.03411	-0.08146	0.19304	Carnitine, O-Acetyl-L-carnitine, Glutamine, Succinic acid
**122**	2.5264	0.02486	-0.08460	0.21357	O-Acetyl-L-carnitine, Glutamine
**123**	2.5431	0.01320	-0.07035	0.21342	O-Acetyl-L-carnitine
**124**	2.5599	0.01715	0.04914	-0.01856	O-Acetyl-L-carnitine
**125**	2.5766	0.04098	-0.00732	0.07555	
**126**	2.5934	0.06350	0.05317	0.10837	Aspartate
**127**	2.6101	0.06535	0.03140	0.13815	Methionine, Carnosine, Anserine, O-Acetyl-L-carnitine, Aspartate
**128**	2.6269	0.06192	0.04646	0.13755	Methionine, Carnosine, Anserine, O-Acetyl-L-carnitine, Aspartate
**129**	2.6436	0.06020	0.10628	0.06919	Methionine, Carnosine, Anserine, O-Acetyl-L-carnitine, Aspartate
**130**	2.6604	0.01979	0.17163	0.05432	Methionine, Carnosine, Anserine, O-Acetyl-L-carnitine, Aspartate
**131**	2.6771	0.00603	0.18801	0.02927	Carnosine, Anserine, O-Acetyl-L-carnitine, Aspartate
**132**	2.6939	0.00585	0.18498	0.03356	Carnosine, Anserine, O-Acetyl-L-carnitine, Aspartate
**133**	2.7106	0.01374	0.18160	0.04975	Carnosine, Anserine, Aspartate
**134**	2.7273	0.03032	0.16782	0.08219	Carnosine, Anserine, Aspartate
**135**	2.7441	0.02448	0.16273	0.09591	Carnosine, Anserine, Aspartate
**136**	2.7608	0.02808	0.15245	0.09870	Anserine, Aspartate
**137**	2.7776	0.04250	0.09992	0.09458	Aspartate

Bucket-numbe r	ppm	PC 1	PC 2	PC 3	Metabolite
**138**	2.7943	0.06448	0.03798	0.07207	Aspartate
**139**	2.8111	0.07385	0.01383	0.03250	Aspartate
**140**	2.8278	0.07735	0.02455	-0.00741	Aspartate
**141**	2.8446	0.07713	0.01206	0.00754	Aspartate
**142**	2.8613	0.07579	0.02404	-0.00170	Aspartate
**143**	2.8781	0.06831	0.02330	-0.05646	Aspartate
**144**	2.8948	0.07811	-0.00690	-0.00593	Aspartate
**145**	2.9116	0.06452	-0.08705	0.04199	Creatinine
**146**	2.9283	0.06711	-0.09623	-0.01036	Creatinine
**147**	2.9451	0.07088	-0.07261	-0.02704	Creatinine
**148**	2.9618	0.06270	-0.04758	-0.03834	Creatinine
**149**	2.9786	0.07472	-0.02013	-0.04130	Creatinine
**150**	2.9953	0.07811	0.00812	-0.03014	Carnosine, Anserine, Creatinine
**151**	3.0120	0.04576	0.15144	0.01788	Tyrosine, Carnosine, Anserine, Creatinine
**152**	3.0288	0.06127	0.05179	-0.02235	Creatine, Tyrosine, Carnosine, Anserine, Creatinine
**153**	3.0455	0.03405	-0.00741	0.01199	Creatine, Tyrosine, Carnosine, Anserine, Creatinine
**154**	3.0623	0.01761	0.15538	0.05290	Tyrosine, Carnosine, Creatinine
**155**	3.0790	0.01330	0.18471	0.04008	Tyrosine, Phenylalanine, Carnosine, Anserine, Creatinine
**156**	3.0958	0.06980	0.08293	0.02018	Phenylalanine, Creatinine
**157**	3.1125	0.01254	0.04766	-0.10778	Phenylalanine, Creatinine
**158**	3.1293	0.05685	-0.03057	0.09053	Phenylalanine, Creatinine
**159**	3.1460	0.07493	0.01904	0.06692	Phenylalanine, O-Acetyl-L-carnitine
**160**	3.1628	0.06005	0.09752	0.02678	Tyrosine, Phenylalanine, Carnosine, Anserine, O-Acetyl-L-carnitine
**161**	3.1795	0.01234	0.18239	0.03374	Tyrosine, Carnosine, Carnitine, Anserine, O-Acetyl-L-carnitine
**162**	3.1963	0.01457	-0.04070	0.23882	Tyrosine, Carnosine, Carnitine, Anserine, O-Acetyl-L-carnitine, Glucose
**163**	3.2130	0.01203	0.18390	0.06346	Tyrosine, Carnosine, Carnitine, Anserine, O-Acetyl-L-carnitine, Glucose
**164**	3.2298	0.01436	0.01106	0.24793	Tyrosine, Carnosine, Carnitine, Anserine, O-Acetyl-L-carnitine, Glucose-6-phosphate, Glucose
**165**	3.2465	0.04507	0.15277	0.03839	Tyrosine, Phenylalanine, Carnosine, Carnitine, Anserine, Glucose-6-phosphate, Glucose
**166**	3.2633	0.03151	-0.06123	0.05448	Betaine, Phenylalanine, Carnosine, Carnitine, Anserine, Glucose-6-phosphate, Glucose
**167**	3.2800	0.06875	0.02962	0.03925	Tryptophan, Phenylalanine, Carnosine, Glucose-6-phosphate, Glucose
**168**	3.2967	0.04714	0.08639	0.11128	Tryptophan, Phenylalanine, Glucose-6-phosphate
**169**	3.3135	0.04149	0.09645	0.10040	Tryptophan, Phenylalanine, Glucose-6-phosphate
**170**	3.3302	0.03688	0.09882	0.08681	Tryptophan
**171**	3.3470	0.04457	-0.04404	0.03689	Glucose
**172**	3.3805	0.07442	0.00716	0.01329	Carnitine
**173**	3.3972	0.07372	-0.00503	0.03620	Carnitine
**174**	3.4140	0.06882	-0.02524	0.01853	Carnitine
**175**	3.4307	0.04764	-0.07431	0.16386	Carnitine
**176**	3.4475	0.06315	-0.05192	0.10544	Tryptophan, Carnitine
**177**	3.4642	0.07276	0.00802	-0.00568	Tryptophan, Carnitine, Glucose-6-phosphate
**178**	3.4810	0.06961	0.00156	0.00002	Tryptophan, Glucose-6-phosphate
**179**	3.4977	0.06783	0.00510	-0.01335	Tryptophan, Glycine, Glucose-6-phosphate

Bucket-numbe r	ppm	PC 1	PC 2	PC 3	Metabolite
**180**	3.5145	0.04638	-0.00813	-0.00528	Tryptophan, Glycine, Glucose-6-phosphate
**181**	3.5312	0.05348	-0.01201	-0.01123	Glycine, Glucose-6-phosphate
**182**	3.5480	0.06481	-0.00977	0.02793	Glycine, Glucose-6-phosphate
**183**	3.5647	0.06839	-0.01923	0.01036	Glycine, Glucose-6-phosphate
**184**	3.5814	0.06272	-0.00117	0.04954	Glycine, Glucose-6-phosphate
**185**	3.5982	0.06988	-0.00621	0.06901	O-Acetyl-L-carnitine, Glycine, Glucose-6-phosphate
**186**	3.6149	0.07665	-0.01803	-0.00311	Valine, O-Acetyl-L-carnitine, Glycine, Glucose-6-phosphate
**187**	3.6317	0.06717	-0.05381	0.07907	O-Acetyl-L-carnitine, Glycine
**188**	3.6484	0.05923	0.01910	0.05835	O-Acetyl-L-carnitine, Glycine
**189**	3.6652	0.07410	0.00548	0.03132	Isoleucine
**190**	3.6819	0.07514	0.01979	0.02106	Isoleucine, Glucose
**191**	3.6987	0.07310	0.00121	0.02824	Glucose-6-phosphate, Glucose
**192**	3.7154	0.07234	-0.00119	0.02363	Leucine, Glucose-6-phosphate, Glucose
**193**	3.7322	0.07429	0.00876	0.03376	Leucine, Glucose-6-phosphate, Glucose
**194**	3.7489	0.07678	0.01918	0.00216	Leucine, Glutamate, Glutamine, Glucose-6-phosphate, Glucose
**195**	3.7657	0.07133	0.00795	-0.00085	Glutamate, Alanine, Glutamine, Glucose
**196**	3.7824	0.05698	0.04062	0.05362	Alanine, Glutamine, Glucose
**197**	3.7992	0.07388	-0.01428	0.03717	Alanine, Glutamine, Glucose
**198**	3.8159	0.07377	-0.00246	0.04707	Alanine, Glucose
**199**	3.8327	0.07441	-0.00771	0.02018	Inosine, Methionine, O-Acetyl-L-carnitine, Aspartate, Glucose
**200**	3.8494	0.07593	-0.00851	0.02348	Inosine, Methionine, O-Acetyl-L-carnitine, Aspartate, Glucose
**201**	3.8661	0.07240	-0.00685	0.01976	Inosine, Methionine, O-Acetyl-L-carnitine, Aspartate, Glucose-6-phosphate, Glucose
**202**	3.8829	0.06972	0.00249	0.04017	Inosine, Methionine, O-Acetyl-L-carnitine, Aspartate, Glucose-6-phosphate, Glucose
**203**	3.8996	0.06816	-0.02448	0.05230	Betaine, Inosine, O-Acetyl-L-carnitine, Aspartate, Glucose-6-phosphate, Glucose
**204**	3.9164	0.07066	0.00473	0.03998	Creatine, Inosine, Aspartate, Creatinine, Glucose-6-phosphate, Glucose
**205**	3.9331	0.03150	-0.04544	-0.00291	Creatine, Inosine, Tyrosine, Aspartate, Creatinine, Glucose-6-phosphate
**206**	3.9499	0.07126	0.00356	0.01153	Inosine, Tyrosine, Aspartate, Creatinine, Glucose-6-phosphate
**207**	3.9666	0.07534	0.00457	0.01299	Tyrosine, Phenylalanine, Aspartate, Glucose-6-phosphate
**208**	3.9834	0.07714	0.00385	0.01408	IMP, Tyrosine, Phenylalanine, Glucose-6-phosphate
**209**	4.0001	0.06509	0.00481	0.03558	IMP, Phenylalanine, Glucose-6-phosphate
**210**	4.0169	0.06287	0.01348	0.05499	IMP, Phenylalanine, Creatinine
**211**	4.0336	-0.01676	0.03453	0.00364	IMP, Tryptophan, Creatinine
**212**	4.0504	0.05712	0.02382	0.05438	IMP, Tryptophan, Creatinine
**213**	4.0671	0.06167	0.05966	0.03935	IMP, Tryptophan, Creatinine
**214**	4.0839	0.07208	0.01487	0.02892	Tryptophan
**215**	4.1006	0.04770	0.08963	0.00396	Lactic acid
**216**	4.1173	0.04713	0.09387	0.00593	Lactic acid
**217**	4.1341	0.04420	0.09958	0.00040	Lactic acid
**218**	4.1508	0.05149	0.09385	-0.00356	Lactic acid
**219**	4.1676	0.07722	0.02056	0.01183	Lactic acid

Bucket-numbe r	ppm	PC 1	PC 2	PC 3	Metabolite
**220**	4.1843	0.07770	0.01512	0.01073	Lactic acid
**221**	4.2011	0.06677	0.04142	0.06058	
**222**	4.2178	0.07096	0.01412	0.05964	
**223**	4.2346	0.07279	0.00354	0.03198	
**224**	4.2513	0.07650	0.00512	0.02205	
**225**	4.2681	0.07769	0.00775	0.02798	Inosine
**226**	4.2848	0.07517	0.01209	0.04897	Inosine
**227**	4.3016	0.07435	0.00554	0.04604	Inosine
**228**	4.3183	0.07599	0.02222	-0.00237	
**229**	4.3351	0.07675	0.02387	-0.02370	
**230**	4.3518	0.07790	0.00725	-0.02627	IMP
**231**	4.3686	-0.03534	-0.00673	-0.07215	IMP
**232**	4.3853	-0.04101	-0.01396	-0.05298	IMP
**233**	4.4020	0.07335	-0.01728	0.01483	
**234**	4.4188	0.07390	-0.00600	0.00688	Inosine
**235**	4.4355	0.07270	0.01580	0.04401	Inosine, Carnosine, Anserine
**236**	4.4523	0.04342	0.15305	0.05385	Inosine, Carnosine, Anserine
**237**	4.4690	0.01177	0.18888	0.03664	Carnosine, Anserine
**238**	4.4858	0.00997	0.18954	0.04331	Carnosine, Anserine
**239**	4.5025	0.00246	0.19131	0.04643	IMP, Carnosine, Carnitine, Anserine
**240**	4.5193	-0.04518	0.10530	0.01018	IMP, Carnosine, Carnitine, Anserine, O-Acetyl-L-carnitine
**241**	4.5360	-0.03360	-0.00907	0.05865	IMP, Carnitine, Anserine, O-Acetyl-L-carnitine
**242**	4.5528	0.01769	-0.09509	0.19171	Carnitine, O-Acetyl-L-carnitine
**243**	4.5695	0.01435	-0.09915	0.20619	Carnitine, O-Acetyl-L-carnitine
**244**	4.5863	0.01673	-0.09336	0.20296	Carnitine, O-Acetyl-L-carnitine
**245**	4.6030	0.02352	-0.05903	0.13797	Carnitine, O-Acetyl-L-carnitine
**246**	4.6198	0.02259	-0.01415	0.03975	Carnitine, Glucose-6-phosphate, Glucose
**247**	4.6365	0.05885	-0.00437	-0.01148	Glucose-6-phosphate, Glucose
**248**	4.6533	0.05430	0.01177	0.01651	Glucose-6-phosphate, Glucose
**249**	4.6700	0.06080	0.00748	0.00616	Glucose-6-phosphate, Glucose

Table S2. VIP scores of the PLS-R (Figure 3).

Bucketnumber	All	Heifer	Cow	All, dry	All, wet
**1**	0.0014	0.0012	0.0025	0.0017	0.0020
**2**	0.0020	0.0018	0.0025	0.0025	0.0029
**3**	0.0012	0.0016	0.0018	0.0013	0.0019
**4**	0.0017	0.0018	0.0040	0.0020	0.0022
**5**	0.0021	0.0023	0.0020	0.0021	0.0020
**6**	0.0018	0.0026	0.0019	0.0022	0.0024
**7**	0.0019	0.0017	0.0025	0.0018	0.0023
**8**	0.0023	0.0029	0.0026	0.0027	0.0028
**9**	0.0019	0.0021	0.0019	0.0020	0.0032
**10**	0.0018	0.0020	0.0029	0.0016	0.0021
**11**	0.0029	0.0033	0.0038	0.0027	0.0040
**12**	0.0033	0.0031	0.0038	0.0042	0.0040
**13**	0.0044	0.0034	0.0068	0.0039	0.0060
**14**	0.0060	0.0058	0.0076	0.0053	0.0069
**15**	0.0066	0.0059	0.0079	0.0058	0.0083
**16**	0.0084	0.0073	0.0100	0.0079	0.0103
**17**	0.0125	0.0117	0.0140	0.0118	0.0154
**18**	0.0183	0.0171	0.0209	0.0168	0.0226
**19**	0.0315	0.0290	0.0357	0.0282	0.0387
**20**	0.0424	0.0395	0.0486	0.0386	0.0520
**21**	0.0663	0.0619	0.0767	0.0614	0.0816
**22**	0.1257	0.1185	0.1462	0.1162	0.1549
**23**	0.2169	0.2037	0.2521	0.2026	0.2659
**24**	0.3066	0.2874	0.3553	0.2871	0.3761
**25**	0.4044	0.3812	0.4619	0.3773	0.4930
**26**	0.6591	0.6294	0.7299	0.5993	0.8021
**27**	1.0341	0.9880	1.1365	0.9254	1.2605
**28**	1.7749	1.7170	1.9133	1.5837	2.1531
**29**	2.5995	2.5401	2.7674	2.3030	3.1491
**30**	1.4272	1.3988	1.5080	1.2313	1.7426
**31**	1.4060	1.3828	1.4785	1.1930	1.7211
**32**	0.7573	0.7384	0.8065	0.6559	0.9231
**33**	1.0686	1.0574	1.1292	0.9035	1.3112
**34**	1.0538	1.0432	1.1097	0.8893	1.2925
**35**	0.1899	0.1829	0.2017	0.1647	0.2325
**36**	0.0599	0.0579	0.0615	0.0535	0.0717
**37**	0.0439	0.0410	0.0477	0.0394	0.0526
**38**	0.0470	0.0449	0.0519	0.0419	0.0574
**39**	0.0839	0.1015	0.0978	0.0765	0.0993
**40**	0.1104	0.1247	0.1308	0.1008	0.1310
**41**	0.1143	0.1034	0.1370	0.1152	0.1406
**42**	0.1249	0.1152	0.1444	0.1245	0.1563
**43**	0.1690	0.1606	0.1862	0.1580	0.2050
**44**	0.2216	0.2128	0.2400	0.2063	0.2682
**45**	0.1728	0.1663	0.1846	0.1611	0.2088
**46**	0.1636	0.1589	0.1716	0.1504	0.1984
**47**	0.1460	0.1400	0.1589	0.1332	0.1791
**48**	0.1391	0.1344	0.1506	0.1298	0.1729
**49**	0.1666	0.1608	0.1865	0.1653	0.2126
**50**	6.2012	6.0202	6.1672	6.4328	4.4700
**51**	6.5921	6.3767	6.6332	6.8888	4.8257
**52**	0.7457	0.7381	0.7813	0.8219	0.8336
**53**	0.2828	0.2691	0.3105	0.2680	0.3537
**54**	0.3027	0.2864	0.3328	0.2831	0.3728
**55**	0.2994	0.2847	0.3280	0.2773	0.3677

Bucketnumber	All	Heifer	Cow	All, dry	All, wet
**56**	0.3230	0.3115	0.3481	0.2969	0.3918
**57**	0.3515	0.3440	0.3686	0.3178	0.4225
**58**	0.3631	0.3576	0.3702	0.3251	0.4344
**59**	1.4350	1.5076	1.3766	1.4251	1.6428
**60**	1.4621	1.5471	1.4004	1.4656	1.6809
**61**	0.4467	0.4520	0.4383	0.4276	0.5344
**62**	0.2415	0.2341	0.2426	0.2225	0.2861
**63**	0.2221	0.2084	0.2424	0.2054	0.2684
**64**	0.1955	0.1851	0.2190	0.1821	0.2360
**65**	0.1059	0.0995	0.1175	0.1000	0.1279
**66**	0.1083	0.1018	0.1204	0.1027	0.1316
**67**	0.1492	0.1450	0.1627	0.1395	0.1831
**68**	0.1958	0.1935	0.2095	0.1818	0.2405
**69**	0.2416	0.2389	0.2507	0.2244	0.2930
**70**	0.3750	0.3720	0.3798	0.3385	0.4486
**71**	0.4846	0.4776	0.4993	0.4336	0.5816
**72**	0.6678	0.6568	0.6998	0.5975	0.8054
**73**	0.9422	0.9305	0.9822	0.8355	1.1339
**74**	1.0179	1.0085	1.0555	0.9001	1.2222
**75**	0.7596	0.7546	0.7801	0.6711	0.9099
**76**	0.5366	0.5324	0.5487	0.4760	0.6422
**77**	0.3168	0.3128	0.3223	0.2872	0.3784
**78**	0.2005	0.1997	0.1975	0.1886	0.2366
**79**	0.1758	0.1745	0.1737	0.1679	0.2064
**80**	0.1760	0.1739	0.1770	0.1652	0.2100
**81**	0.1878	0.1835	0.1928	0.1737	0.2250
**82**	0.2037	0.1956	0.2117	0.1890	0.2412
**83**	0.2583	0.2496	0.2646	0.2361	0.3045
**84**	0.4097	0.4002	0.4160	0.3669	0.4841
**85**	0.5777	0.5661	0.5875	0.5124	0.6863
**86**	0.9106	0.8812	0.9833	0.8205	1.0929
**87**	0.4711	0.4563	0.4866	0.4248	0.5572
**88**	0.3489	0.3350	0.3647	0.3176	0.4129
**89**	0.3406	0.3289	0.3566	0.3096	0.4063
**90**	0.3688	0.3648	0.3733	0.3344	0.4405
**91**	0.4041	0.4051	0.4016	0.3663	0.4815
**92**	0.4464	0.4447	0.4437	0.4040	0.5317
**93**	0.4611	0.4599	0.4545	0.4144	0.5493
**94**	0.4765	0.4743	0.4660	0.4263	0.5625
**95**	0.4797	0.4808	0.4664	0.4261	0.5685
**96**	0.4810	0.4780	0.4674	0.4269	0.5698
**97**	0.5042	0.4787	0.5106	0.4571	0.5911
**98**	0.5954	0.5030	0.6726	0.5695	0.6857
**99**	1.3726	1.3280	1.2331	1.2640	1.5436
**100**	0.6269	0.4870	0.7642	0.6115	0.7282
**101**	0.3266	0.2588	0.4041	0.3167	0.3801
**102**	0.1622	0.1507	0.1746	0.1534	0.1904
**103**	0.1669	0.1578	0.1772	0.1556	0.1984
**104**	0.2075	0.1988	0.2169	0.1945	0.2475
**105**	0.2619	0.2611	0.2566	0.2470	0.3100
**106**	0.3039	0.3035	0.2952	0.2858	0.3584
**107**	0.3545	0.3505	0.3472	0.3289	0.4187
**108**	0.3158	0.3024	0.3271	0.2904	0.3763
**109**	0.2814	0.2717	0.2870	0.2594	0.3334
**110**	0.2460	0.2398	0.2465	0.2278	0.2898
**111**	0.4933	0.4855	0.4748	0.4130	0.5899
**112**	0.6486	0.6568	0.6158	0.5211	0.7792

Bucketnumber	All	Heifer	Cow	All, dry	All, wet
**113**	0.3566	0.3519	0.3284	0.3119	0.4138
**114**	0.1878	0.1751	0.1909	0.1806	0.2103
**115**	0.9338	1.0998	1.1116	1.0898	0.9897
**116**	0.2253	0.1606	0.2165	0.2321	0.2186
**117**	0.6242	0.3782	0.7095	0.6290	0.6876
**118**	0.8023	0.5148	1.0370	0.8015	0.9223
**119**	0.3951	0.2826	0.4996	0.3978	0.4422
**120**	0.2007	0.1742	0.2209	0.2072	0.2072
**121**	0.0992	0.0954	0.1047	0.1078	0.0980
**122**	0.1174	0.1144	0.1141	0.1427	0.0973
**123**	0.1070	0.0566	0.1332	0.1107	0.1164
**124**	0.1133	0.0480	0.1355	0.1191	0.1254
**125**	0.1607	0.1614	0.1144	0.1888	0.1442
**126**	0.0535	0.0536	0.0576	0.0547	0.0597
**127**	0.0788	0.0848	0.0730	0.0780	0.0908
**128**	0.2522	0.2594	0.2316	0.2317	0.3059
**129**	0.3929	0.3911	0.4041	0.3557	0.5008
**130**	0.4022	0.4658	0.4111	0.4115	0.4823
**131**	0.9829	0.9706	0.9855	0.8854	1.2078
**132**	1.1934	1.1862	1.1218	1.0681	1.4715
**133**	0.5994	0.6057	0.5579	0.5526	0.7743
**134**	0.3076	0.3408	0.2988	0.2921	0.3848
**135**	0.3044	0.4089	0.2852	0.2987	0.3606
**136**	0.2029	0.2756	0.1939	0.2036	0.2286
**137**	0.1099	0.1439	0.1036	0.1139	0.1110
**138**	0.1234	0.1376	0.1182	0.1248	0.1346
**139**	0.1207	0.1197	0.1225	0.1153	0.1398
**140**	0.1196	0.1123	0.1308	0.1060	0.1463
**141**	0.1628	0.1572	0.1700	0.1463	0.1973
**142**	0.0898	0.0851	0.0950	0.0823	0.1148
**143**	0.1708	0.1506	0.1580	0.1559	0.2139
**144**	0.1227	0.1174	0.1290	0.1053	0.1505
**145**	0.0535	0.0438	0.0563	0.0511	0.0610
**146**	0.0925	0.0829	0.0888	0.0813	0.1083
**147**	0.1257	0.1188	0.1230	0.1075	0.1499
**148**	0.0854	0.0674	0.0901	0.0758	0.1069
**149**	0.1615	0.1391	0.1791	0.1459	0.1993
**150**	0.3001	0.2812	0.3225	0.2742	0.3690
**151**	0.5874	0.5701	0.6628	0.5325	0.8025
**152**	0.5131	0.4958	0.5873	0.5018	0.6704
**153**	3.5470	3.4160	4.1302	3.8854	2.6973
**154**	0.5427	0.5038	0.5431	0.5324	0.6649
**155**	0.5105	0.5090	0.5145	0.4573	0.6641
**156**	0.1651	0.1643	0.1717	0.1508	0.2053
**157**	0.6286	0.6354	0.9185	0.6394	0.6237
**158**	0.2455	0.2634	0.2323	0.2830	0.2437
**159**	0.2318	0.2287	0.2390	0.2135	0.2772
**160**	0.2548	0.2352	0.2638	0.2459	0.3051
**161**	0.7764	0.7999	0.9160	0.7461	0.9720
**162**	1.5939	1.6721	0.6539	1.5088	1.6112
**163**	1.2932	1.3635	1.2320	1.1969	1.6343
**164**	2.9767	3.1640	2.3517	2.8891	2.9997
**165**	1.4038	1.3812	1.4888	1.2890	1.8828
**166**	2.6467	2.7036	2.5396	2.7493	2.1347
**167**	0.9176	0.9701	0.8836	0.9126	1.0349
**168**	0.4796	0.6390	0.4127	0.5078	0.4720
**169**	0.4961	0.6495	0.4446	0.5275	0.4555

Bucketnumber	All	Heifer	Cow	All, dry	All, wet
**170**	0.2352	0.3208	0.2218	0.2609	0.2093
**171**	0.1917	0.2002	0.1777	0.1907	0.1952
**172**	0.3252	0.3366	0.3047	0.3082	0.3863
**173**	0.4294	0.4277	0.4209	0.4051	0.5117
**174**	1.1598	1.1700	1.1390	1.1082	1.3349
**175**	1.3103	1.1520	1.2670	1.3110	1.3991
**176**	0.8230	0.7871	0.7949	0.8010	0.9286
**177**	0.7675	0.7749	0.7534	0.7228	0.9241
**178**	0.8725	0.8900	0.8410	0.8349	1.0639
**179**	0.9160	0.9439	0.8776	0.8832	1.1194
**180**	0.6294	0.6422	0.6694	0.6545	0.7025
**181**	0.8664	0.8944	0.8759	0.9085	0.9949
**182**	0.8261	0.9074	0.6989	0.8702	0.9245
**183**	1.4169	1.4821	1.2804	1.4268	1.6526
**184**	1.4304	1.6228	1.1170	1.4837	1.5956
**185**	1.0035	1.1254	0.8273	1.0396	1.1242
**186**	0.5830	0.5855	0.5609	0.5492	0.6758
**187**	0.5659	0.5953	0.5089	0.5615	0.6127
**188**	1.0823	1.3721	1.1352	1.2319	1.1694
**189**	1.1259	1.1867	1.0783	1.0977	1.3124
**190**	1.1667	1.2442	1.1338	1.1340	1.3843
**191**	1.4070	1.4449	1.3131	1.3252	1.6669
**192**	1.6320	1.6744	1.5219	1.5373	1.9496
**193**	1.9000	1.9349	1.7934	1.7750	2.2828
**194**	1.9224	1.9159	1.9172	1.7514	2.3506
**195**	1.7661	1.7254	1.8152	1.6564	2.0835
**196**	2.3672	2.4332	2.6116	2.2942	2.7609
**197**	2.0371	2.1456	1.9684	1.9880	2.3322
**198**	1.7754	1.8313	1.6648	1.7193	2.0699
**199**	1.9072	1.9280	1.8235	1.8382	2.2190
**200**	1.7526	1.7698	1.6787	1.6687	2.0660
**201**	1.6802	1.7342	1.5530	1.6455	1.9993
**202**	1.2523	1.3269	1.1150	1.2151	1.4953
**203**	1.6635	1.7341	1.4680	1.6073	1.9397
**204**	1.1754	1.2452	1.0264	1.1512	1.4052
**205**	2.4024	2.3239	2.8716	2.6265	1.9577
**206**	1.1758	1.2079	1.1039	1.1318	1.4370
**207**	0.8328	0.8446	0.8010	0.7768	1.0048
**208**	0.8623	0.8673	0.8443	0.7903	1.0350
**209**	1.4222	1.5181	1.2944	1.3842	1.6618
**210**	1.5537	1.7130	1.3594	1.5158	1.7809
**211**	1.3748	1.3918	1.4236	1.4071	1.3348
**212**	0.5470	0.6155	0.5181	0.5408	0.5918
**213**	0.8528	0.8655	0.8851	0.8732	0.9588
**214**	0.2636	0.2648	0.2629	0.2849	0.2930
**215**	0.6417	0.6494	0.6542	0.7012	0.6293
**216**	1.6718	1.7085	1.6810	1.8216	1.7024
**217**	1.5300	1.5299	1.5602	1.6507	1.4348
**218**	0.5823	0.5808	0.6045	0.6350	0.6156
**219**	0.3118	0.3050	0.3202	0.2990	0.3668
**220**	0.3744	0.3585	0.3927	0.3552	0.4369
**221**	0.3037	0.3276	0.2945	0.3045	0.3323
**222**	0.2220	0.2283	0.2108	0.2228	0.2500
**223**	0.2176	0.2144	0.2194	0.2169	0.2573
**224**	0.2797	0.2773	0.2787	0.2644	0.3358
**225**	0.3642	0.3648	0.3527	0.3513	0.4292
**226**	0.4777	0.4735	0.4566	0.5079	0.5080

Bucketnumber	All	Heifer	Cow	All, dry	All, wet
**227**	0.2885	0.2844	0.2984	0.2776	0.3394
**228**	0.2621	0.2545	0.2806	0.2527	0.3148
**229**	0.2656	0.2650	0.2780	0.2501	0.3236
**230**	0.2242	0.2209	0.2363	0.2070	0.2738
**231**	0.4901	0.4895	0.4916	0.5197	0.4807
**232**	0.4971	0.4841	0.4954	0.5226	0.4990
**233**	0.1135	0.1026	0.1265	0.1154	0.1371
**234**	0.1228	0.1155	0.1340	0.1219	0.1469
**235**	0.2581	0.2570	0.2631	0.2876	0.2677
**236**	0.3442	0.3446	0.3560	0.3790	0.3916
**237**	0.5245	0.5304	0.5566	0.4750	0.6675
**238**	0.6828	0.7014	0.6526	0.6159	0.8615
**239**	0.4845	0.5544	0.4363	0.4419	0.5184
**240**	0.6711	0.7415	0.6152	0.6780	0.6636
**241**	0.2847	0.3111	0.2681	0.2946	0.2935
**242**	0.0806	0.0633	0.0884	0.0849	0.0885
**243**	0.1332	0.0992	0.1097	0.1343	0.1422
**244**	0.1148	0.0852	0.1129	0.1227	0.1227
**245**	0.0645	0.0502	0.1096	0.0896	0.0741
**246**	0.0563	0.0492	0.1152	0.0872	0.0640
**247**	0.3470	0.3451	0.3885	0.3490	0.4182
**248**	0.2670	0.2626	0.2996	0.2910	0.3054
**249**	0.4525	0.4563	0.4715	0.4641	0.5358

Table S3. Generalized linear mixed-effect models for each metabolite.

Metabolite	Model[1]	Name	Coefficient	Std	*p*-value	R^2
		**Amino acids**				
**Alanine**	1	Intercept	0.070	0.004	< 0.001	0.77
		Aging time	-0.002	5.62 × 10^{-5}	< 0.001	
		Cattle (C) * Aging time	2.23 × 10^{-4}	8.11 × 10^{-5}	0.005	
	2	Intercept	0.073	0.004	< 0.001	0.58
**Aspartate**	1	Intercept	4.565	1.344	< 0.001	0.65
		Cattle (C)	4.229	1.936	0.029	
		Aging time	-0.076	0.008	< 0.001	
		Cattle (C) * Aging time	-0.147	0.015	< 0.001	
	2	Intercept	12.526	2.077	< 0.001	0.61
**Glutamate**	1	Intercept	0.480	0.040	< 0.001	0.63
		Aging time	-0.012	0.001	< 0.001	
		Aging type (W)	-0.068	0.022	0.002	
		Cattle (C) * Aging time	-0.004	0.001	< 0.001	
		Aging time * Aging type (W)	0.002	0.001	0.024	
	2	Intercept	0.784	0.072	< 0.001	0.68
**Glutamine**	1	Intercept	0.042	0.002	< 0.001	0.71
		Aging time	-0.001	4.11 × 10^{-5}	< 0.001	
		Cattle (C) * Aging time	-1.44 × 10^{-4}	6.14 × 10^{-5}	0.019	
	2	Intercept	0.058	0.009	< 0.001	0.76
**Glycine**	1	Intercept	0.608	0.031	< 0.001	0.76
		Aging time	-0.012	0.001	< 0.001	
		Cattle (C) * Aging time	-0.002	0.001	0.023	
	2	Intercept	0.782	0.060	< 0.001	0.72
**Isoleucine**	1	Intercept	7.385	0.440	< 0.001	0.53
		Aging time	-0.221	0.011	< 0.001	
		Aging type (W)	-1.460	0.388	< 0.001	
		Aging time * Aging type (W)	0.049	0.015	< 0.001	
	2	Intercept	26.592	3.053	< 0.001	0.74
**Leucine**	1	Intercept	2.504	0.166	< 0.001	0.52
		Aging time	-0.074	0.004	< 0.001	
		Aging type (W)	-0.456	0.139	0.001	
		Aging time * Aging type (W)	0.015	0.005	0.003	
	2	Intercept	11.046	1.417	< 0.001	0.75
**Methio-nine**	1	Intercept	0.632	0.053	< 0.001	0.87
		Aging time	-0.013	4.00 × 10^{-4}	< 0.001	
	2	Intercept	1.107	0.409	0.008	0.98
**Phenyl-alanine**	1	Intercept	2.619	0.088	< 0.001	0.81
		Aging time	-0.069	0.002	< 0.001	
		Aging type (W)	-0.303	0.066	< 0.001	
		Aging time * Aging type (W)	0.008	0.003	0.004	
	2	Intercept	3.608	0.117	< 0.001	0.48
	1	Intercept	48.433	1.756	< 0.001	0.81

Metabolite	Model[1]	Name	Coefficient	Std	*p*-value	R^2
**Trypto-phan**		Aging time	-1.181	0.038	< 0.001	
		Aging type (W)	-5.266	1.186	< 0.001	
		Aging time * Aging type (W)	0.128	0.051	0.012	
	2	Intercept	58.995	1.998	< 0.001	0.31
**Tyrosine**	1	Intercept	4.994	0.259	< 0.001	0.65
		Aging time	-0.142	0.006	< 0.001	
		Aging type (W)	-0.953	0.202	< 0.001	
		Aging time * Aging type (W)	0.030	0.008	< 0.001	
	2	Intercept	12.056	1.243	< 0.001	0.75
**Valine**	1	Intercept	2.734	0.145	< 0.001	0.54
		Aging time	-0.083	0.004	< 0.001	
		Aging type (W)	-0.568	0.137	< 0.001	
		Aging time * Aging type (W)	0.019	0.005	< 0.001	
	2	Intercept	8.634	0.840	< 0.001	0.73
		**Nucleotides**				
**Hypoxan-thine**	1	Intercept	0.276	0.013	< 0.001	0.72
		Cattle (C)	0.140	0.017	< 0.001	
		Aging time	-0.008	3.98×10^{-4}	< 0.001	
		Aging type (W)	-0.075	0.011	< 0.001	
		Cattle (C) * Aging time	-0.004	0.001	< 0.001	
		Aging time * Aging type (W)	0.002	4.69×10^{-4}	< 0.001	
	2	Intercept	0.765	0.083	< 0.001	0.58
**IMP**	1	Aging time	0.024	0.001	< 0.001	0.67
		Aging type (W)	-0.052	0.016	< 0.001	
		Cattle (C) * Aging time	-0.015	0.001	< 0.001	
		Aging time * Aging type (W)	0.008	0.001	< 0.001	
	2	Intercept	0.087	0.045	< 0.001	0.78
**Inosine**	1	Intercept	0.618	0.062	< 0.001	0.79
		Cattle (C)	0.217	0.090	0.016	
		Aging time	-0.010	0.001	< 0.001	
		Aging type (W)	0.113	0.009	< 0.001	
	2	Intercept	1.486	0.096	< 0.001	0.72
		**Acids**				
**Acetic acid**	1	Intercept	4.636	0.365	< 0.001	0.72
		Cattle (C)	-1.151	0.518	0.027	
		Aging time	-0.111	0.005	< 0.001	
		Cattle (C) * Aging time	0.028	0.006	< 0.001	
	2	Intercept	7.402	0.892	< 0.001	0.79
		Cattle (C)	-2.794	1.296	0.033	
**Fumaric acid**	1	Intercept	14.414	1.474	< 0.001	0.30
		Aging time	-0.348	0.034	< 0.001	
		Cattle (C) * Aging time	0.161	0.049	< 0.001	
	2	Intercept	39.823	6.911	< 0.001	0.51
		Cattle (C)	-20.483	9.85	0.040	

Metabolite	Model[1]	Name	Coefficient	Std	p-value	R^2
**Lactic acid**	1	Intercept	2.00×10^{-4}	3.19×10^{-6}	< 0.001	0.43
		Aging time	-1.97×10^{-6}	1.78×10^{-7}	< 0.001	
		Cattle (C) * Aging time	-1.05×10^{-6}	1.23×10^{-7}	< 0.001	
		Aging time * Aging type (W)	1.94×10^{-6}	1.26×10^{-7}	< 0.001	
	2	Intercept	2.08×10^{-4}	3.36×10^{-6}	< 0.001	0.00
**Succinic acid**	1	Intercept	3.411	0.414	< 0.001	0.66
		Cattle (C)	-2.229	0.595	< 0.001	
		Aging time	-0.054	0.005	< 0.001	
		Cattle (C) * Aging time	0.045	0.007	< 0.001	
	2	Intercept	8.854	1.318	< 0.001	0.72
		Cattle (C)	-6.704	1.878	< 0.001	
		**Sugars**				
**α Glucose**	1	Intercept	0.123	0.005	< 0.001	0.60
		Aging time	-0.003	1.13×10^{-4}	< 0.001	
	2	Intercept	0.250	0.023	< 0.001	0.40
**β Glucose**	1	Intercept	0.047	0.003	< 0.001	0.47
		Aging time	-0.001	5.83×10^{-5}	< 0.001	
	2	Intercept	0.086	0.010	< 0.001	0.26
**Glucose-6-phosphate**	1	Intercept	0.312	0.042	< 0.001	0.57
		Cattle (C) * Aging time	-0.002	0.001	0.017	
	2	Intercept	0.533	0.076	< 0.001	0.40
		**Further compounds**				
**Anserine**	1	Intercept	0.376	0.156	0.017	0.94
		Aging time	-0.004	2.91×10^{-4}	< 0.001	
**Betaine**	1	Intercept	0.039	0.003	< 0.001	0.77
		Aging time	-0.001	3.31×10^{-5}	< 0.001	
		Cattle (C) * Aging time	-2.13×10^{-4}	4.88×10^{-5}	< 0.001	
		Aging time * Aging type (W)	4.62×10^{-5}	1.64×10^{-5}	0.005	
	2	Intercept	0.054	0.004	< 0.001	0.85
**Carnitine**	1	Intercept	0.060	0.017	< 0.001	0.94
		Cattle (C)	0.162	0.024	< 0.001	
		Aging time	-0.001	7.17×10^{-5}	< 0.001	
		Cattle (C) * Aging time	-0.002	1.92×10^{-4}	< 0.001	
		Aging time * Aging type (W)	0.001	5.20×10^{-5}	< 0.001	
	2	Intercept	0.052	0.013	< 0.001	0.97
		Cattle (C)	0.162	0.019	< 0.001	
**Carnosine**	1	Aging time	-2.22×10^{-5}	2.66×10^{-6}	< 0.001	0.99
**Creatine**	1	Intercept	0.001	5.00×10^{-5}	< 0.001	0.63
		Aging time	-5.07×10^{-6}	7.45×10^{-7}	< 0.001	
		Cattle (C) * Aging time	-4.29×10^{-6}	1.08×10^{-6}	< 0.001	
		Aging time * Aging type (W)	6.21×10^{-6}	4.40×10^{-7}	< 0.001	
	2	Intercept	9.98×10^{-4}	1.51×10^{-5}	< 0.001	0.14
		Cattle (C)	9.90×10^{-5}	2.29×10^{-5}	< 0.001	
**Creatinine**	1	Intercept	0.374	0.035	< 0.001	0.51
		Aging time	-0.006	0.001	< 0.001	

Metabolite	Model[1]	Name	Coefficient	Std	*p*-value	R^2
		Aging type (W)	-0.051	0.016	0.002	
		Cattle (C) * Aging time	-0.002	0.001	0.032	
		Aging time * Aging type (W)	0.003	0.001	< 0.001	
	2	Intercept	0.679	0.076	< 0.001	0.61
**Niacin-amide**	1	Intercept	5.408	0.221	< 0.001	0.63
		Cattle (C)	1.086	0.304	< 0.001	
		Aging time	-0.072	0.006	< 0.001	
		Aging type (W)	-0.718	0.152	< 0.001	
		Cattle (C) * Aging time	-0.047	0.007	< 0.001	
		Aging time * Aging type (W)	0.027	0.007	< 0.001	
	2	Intercept	6.078	0.395	< 0.001	0.69
		Cattle (C)	1.594	0.582	0.007	
**O-Acetyl-L-carnitine**	1	Intercept	0.278	0.103	0.007	0.87
		Cattle (C)	0.708	0.153	< 0.001	
		Cattle (C) * Aging time	0.016	0.002	< 0.001	
	2	Intercept	0.488	0.115	< 0.001	0.84
		Cattle (C)	0.678	0.170	< 0.001	

[1] model 1 – calculated with all samples; model 2 – calculated with samples of day 0

Cattle – group of cows (C) and heifers (H); C is used as reference

Aging type – group of wet-aged (W) and dry-aged (D) samples; W is used as reference
